# Haemodynamic Optimization by Oesophageal Doppler and Pulse Power Wave Analysis in Liver Surgery: A Randomised Controlled Trial

**DOI:** 10.1371/journal.pone.0132715

**Published:** 2015-07-17

**Authors:** Aarne Feldheiser, Velizara Pavlova, Karin Weimann, Oliver Hunsicker, Martin Stockmann, Mandy Koch, Alexander Giebels, Klaus-Dieter Wernecke, Claudia D. Spies

**Affiliations:** 1 Department of Anaesthesiology and Intensive Care Medicine, Campus Charité Mitte and Campus Virchow-Klinikum, Charité - University Medicine Berlin, Berlin, Germany; 2 Department of General-, Visceral- and Transplantation Surgery, Charité - University Medicine Berlin, Campus Virchow-Klinikum, Berlin, Germany; 3 Charité - University Medicine Berlin and SOSTANA GmbH Berlin, Berlin, Germany; University Hospital Oldenburg, GERMANY

## Abstract

Liver surgery is still associated with a high rate of morbidity and mortality. We aimed to compare different haemodynamic treatments in liver surgery. In a prospective, blinded, randomised, controlled pilot trial patients undergoing liver resection were randomised to receive haemodynamic management guided by conventional haemodynamic parameters or by oesophageal Doppler monitor (ODM, CardioQ-ODM) or by pulse power wave analysis (PPA, LiDCOrapid) within a goal-directed algorithm adapted for liver surgery. The primary endpoint was stroke volume index before intra-operative start of liver resection. Secondary endpoints were the haemodynamic course during surgery and postoperative pain levels. Due to an unbalance in the extension of the surgical procedures with a high rate of only minor procedures the conventional group was dropped from the analysis. Eleven patients in the ODM group and 10 patients in the PPA group were eligible for statistical analysis. Stroke volume index before start of liver resection was 49 (37; 53) ml/m^2^ and 48 (41; 56) ml/m^2^ in the ODM and PPA group, respectively (p=0.397). The ODM guided group was haemodynamically stable as shown by ODM and PPA measurements. However, the PPA guided group showed a significant increase of pulse-pressure-variability (p=0.002) that was not accompanied by a decline of stroke volume index displayed by the PPA (p=0.556) but indicated by a decline of stroke volume index by the ODM (p<0.001). The PPA group had significantly higher postoperative pain levels than the ODM group (p=0.036). In conclusion, goal-directed optimization by ODM and PPA showed differences in intraoperative cardiovascular parameters indicating that haemodynamic optimization is not consistent between the two monitors.

**Trial Registration:**

ISRCTN.com ISRCTN64578872

## Introduction

The advances in surgical techniques and instruments and the improved knowledge of liver anatomy have led to a decrease in perioperative morbidity and mortality after liver resections over the past two decades [1, 2]. However, perioperative morbidity and mortality is still relevant in liver surgery and the amount of intraoperative blood losses and transfused blood units are key determinants of postoperative morbidity and mortality of patients after liver resections [3]. From the anesthesiological view, a possibility to reduce intraoperative blood losses is to guide therapy towards a low central venous pressure (CVP) between 2 to 5 mmHg or below 5 mmHg, especially during parenchymal incision and resection [4–6]. Lowering the CVP is pathophysiologically based on the mechanism to reduce backflow of blood from the right atrium through the liver veins to the liver resection area. This approach has been shown to improve postoperative outcome and reduce hospital length of stay [5–7].

A low CVP is reached by a restrictive intraoperative fluid and volume therapy and the administration of vasodilatative drugs such as nitroglycerine [6, 8]. The restrictive approach is based on a very low fixed infusion rate of crystalloid solution and additional fluids guided by conventional haemodynamic parameters such as arterial blood pressure (ABP), heart rate (HR) and urine output (UO) [8]. However, this approach entails the risk of occult hypovolaemia as conventional haemodynamic parameters are not sensitive enough to detect early hypovolaemia and tissue hypoperfusion [9–12]. The misjudgement and a delayed therapy of hypovolaemia lead to a deteriorated organ function in the postoperative course, [13–15] which is associated with a prolonged hospital stay, increased costs and higher mortality [16, 17].

An intraoperative goal-directed therapy based on advanced haemodynamic monitoring could reduce the postoperative morbidity and hospital length of stay in different types of non-cardiac surgery. In most studies volume therapy was guided to optimize stroke volume based on the Frank-Starling mechanism [18, 19]. In abdominal surgery, the published haemodynamic algorithms are aimed at maintaining normovolaemia while in liver surgery this approach would lead to an increased risk of bleeding especially during parenchymal resection and result in an increase of postoperative morbidity. Hence, a goal-directed algorithm for liver surgery needs to be adapted for the demands of liver surgery and should include maintenance of circulatory flow with a restrictive fluid and volume approach.

Different advanced haemodynamic monitors based on different methods to measure stroke volume such as ultrasound, pulse power wave analysis or thermodilution could potentially guide such an algorithm. However, in absence of a gold standard a controversy still exists about the use of different haemodynamic monitors to guide an intraoperative haemodynamic management, especially in liver surgery.

Consequently, the aims of the current pilot study were (1) to compare intraoperative haemodynamics and the postoperative clinical course in patients undergoing liver resection whose haemodynamic management was guided by conventional haemodynamic parameters or by oesophageal Doppler monitor (ODM, CardioQ-ODM) or pulse power wave analysis (PPA, LiDCOrapid) within a haemodynamic algorithm adapted for liver surgery; (2) to compare the ODM and PPA in regard to agreement and trending during the course of surgery.

## Methods

This was a prospective, single-center, blinded, parallel-group, three-armed randomised controlled trial as a pilot study. Registration of the study was performed internationally (International Standard Randomised Controlled Trial Number Register: ISRCTN64578872, applied 10^th^ March 2009, assigned on 3^rd^ December 2009, principal investigator: Claudia Spies) and ethical vote was attained from the ethical commission (Ethics Committee of Charité—University Medicine Berlin approved on the 22^nd^ January 2009, No. EA 1/004/09). All patients gave written informed consent. Eligible patients were aged at least 18 years and undergoing elective liver resection (hemihepatectomy or extended liver resection) at Charité—University Medicine Berlin, Campus Virchow- Klinikum, Berlin, Germany. Exclusion criteria were age less than 18 years, pregnancy or lactation, being unable or unwilling to give written consent to data storage and processing within clinical studies, member of staff of the Charité, simultaneous participation in another study, accommodation in an institution due to an official or judicial order, advanced disease, or operations within the last two months of the oesophagus of nasopharyngeal cavity, history of bleeding tendency, neurological or psychiatric disease, unclear history of alcohol related disorder, chronic heart failure class IV according to the New York Heart Association (NYHA), American Society of Anaesthesiologists (ASA) classification over III, renal insufficiency with dependency on haemodialysis, pulmonary oedema in the pre-operative chest X-ray, allergy to gelatine, history of intracranial haemorrhage within one year before participation in the study before inclusion.

### Study design: planning, randomization and blinding

As there were no preliminary data in the literature, describing patients being treated within a goal-directed haemodynamic algorithm for liver surgery this study was planned as a pilot study. Before admission to the operating room the patients were randomly assigned to the study groups in a 1:1:1 ratio for parallel arms according to a randomization list created by a biostatistician in blocks of six patients with sealed envelopes. The sealed envelopes were in compulsory order, gave information about the group allocation, had to be filled out and signed by the study personnel and re-sealed thereafter. There was no stratification used in this study. The patients were blinded to their allocated study intervention.

### Clinical pathway

The patients were evaluated, prepared for the operation and treated according to the published and certified standards (according to DIN EN ISO 9001) of the Department of Anaesthesiology and Intensive Care Medicine, Campus Virchow-Klinikum and Campus Charité Mitte, Charité—University Medicine Berlin, Germany.

### Study interventions

The patients were randomly assigned to receive intraoperative haemodynamic management guided by (1) conventional haemodynamic parameters or by (2) oesophageal Doppler monitor (ODM, CardioQ-ODM) or by (3) pulse power wave analysis (PPA, LiDCOrapid). In each of the three allocation groups both the ODM and PPA were established.

In the conventional group (1) measurements of the ODM and PPA were performed by the study personnel while the monitors were blinded to the treating anesthesiological and surgical team. All haemodynamic treatments in the conventional group were performed according to the clinical decision of the anaesthetist in charge of the patient. Briefly, the aim was a restrictive administration of intravenous infusions to lower the CVP towards a value of zero. Based on conventional haemodynamic parameters such as arterial blood pressure (ABP), norepinephrine dosage, heart rate (HR) and urine output (UO) additional fluid and volume administration was performed to avoid severe tissue hypoperfusion. ABP was maintained by continuous or bolus administration of norepinephrine.

In the ODM group (2) the CardioQ-ODM was shown to the treating personnel and the goal-directed algorithm was performed according to the values measured by the ODM, while blinded measurements of the PPA were performed by study personnel.

In the PPA group (3) the LiDCOrapid was shown to the treating personnel and the algorithm was performed according to the values measured by the PPA, while blinded measurements of the ODM were performed by study personnel.

### Patients

Fifty patients were assessed for eligibility between March 2009 and August 2010. A total of 41 patients were randomly assigned to the study groups.

Two patients in the PPA group and two patients in the conventional group did not receive the allocated operation as for three patients surgery was cancelled and one patient withdrew his participation prior to surgery. Two patients in the ODM and one patient in the PPA group had to be taken out of the intention-to-treat group as the surgical procedure was changed after laparotomy. In these patients a biliodigestive anastomosis (n = 1), a pylorus-preserving pancreaticoduodenectomy (n = 1) and a diagnostic lymphonodectomy (n = 1) were performed.

The conventional group showed a higher number of patients who had to be excluded for serious reasons (n = 4). In these patients an explorative laparotomy (n = 3) and a diagnostic lymphonodectomy (n = 1) were performed. Furthermore, there were less extended surgical procedures in the conventional group. An additional recruitment for patients with more extended procedures resulted in differences between the additional and randomized controls and couldn’t settle the unbalance. Therefore, we decided to drop those patients.

**Fig 1:**
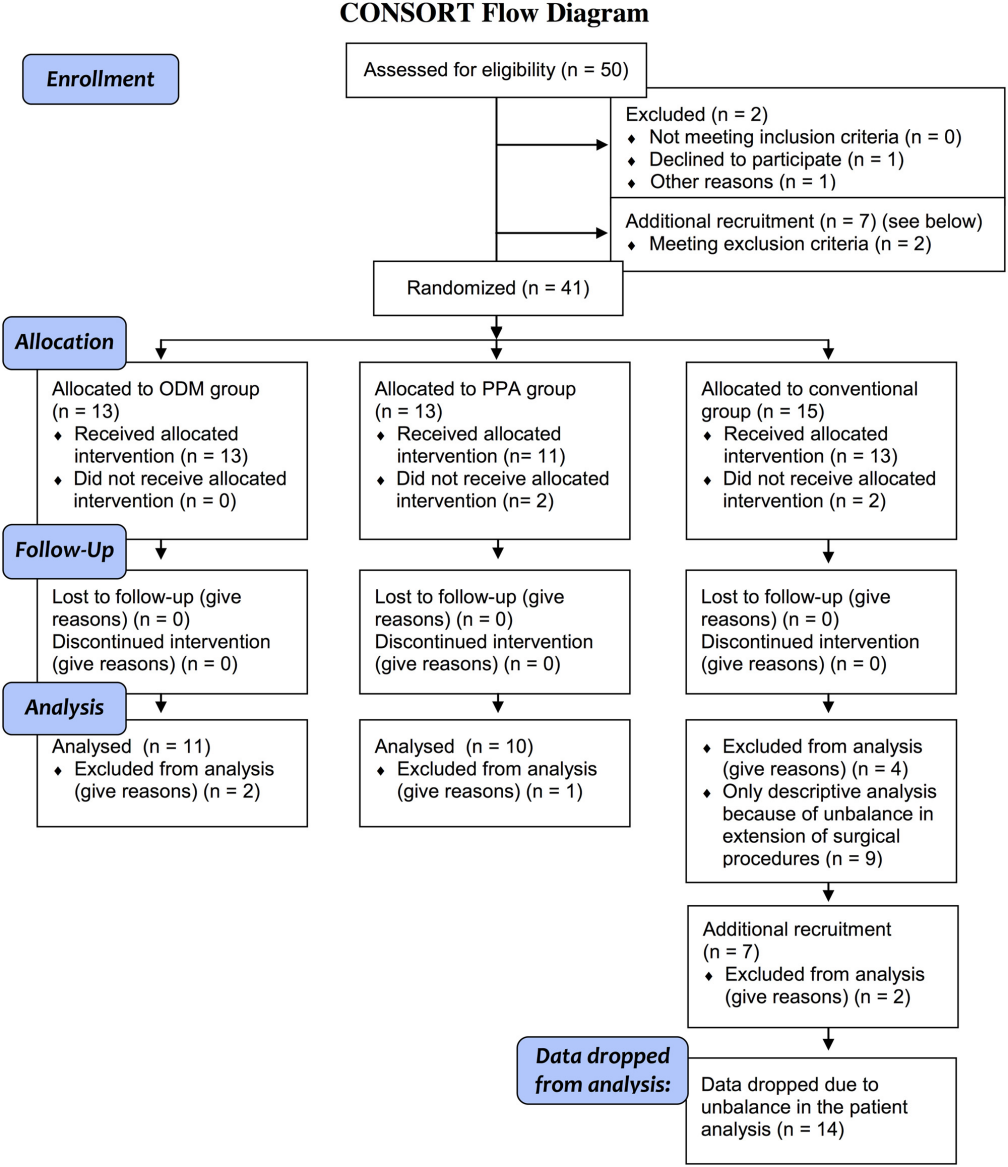
CONSORT Flow Diagram of the study.

In total, 21 patients (ODM group n = 11 and PPA group n = 10) were included in the statistical analysis for primary and secondary endpoints (Fig 1 CONSORT Flow Diagram of the study).

### Haemodynamic protocol and data collection

**Fig 2:**
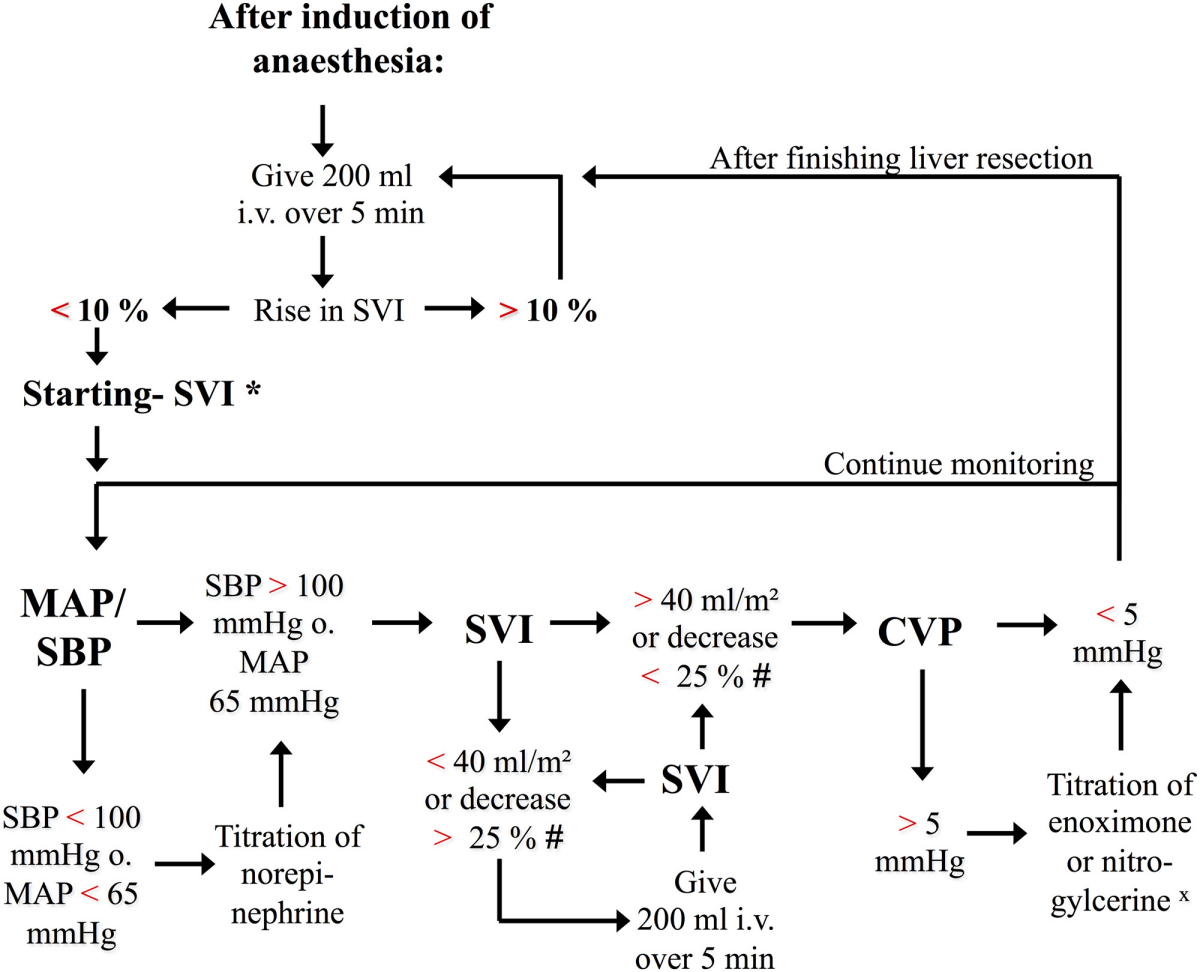
Goal-directed haemodynamic algorithm for liver surgery. **Haemodynamic algorithm-abbreviations:** SVI: stroke volume index [ml/m²], MAP: mean arterial pressure [mmHg], SBP: systolic blood pressure [mmHg], CVP: central venous pressure [mmHg]. **Guidelines on the use of the algorithm:** After induction of anaesthesia and implementation of advanced haemodynamic monitoring a fluid challenge with 200ml of a colloid solution is performed. If stroke SVI increases by more than 10% a further fluid challenge is performed up to the point SVI is not increasing anymore. The value of SVI after the last fluid challenge is defined as Starting-SVI. After determining the Starting-SVI the second time period of the haemodynamic algorithm starts up to the point the liver resection area is surgically sealed. During that period arterial blood pressure is maintained by titration of continuous administration of norepinephrine to reach a systolic blood pressure (SBP) of more than 100mmHg and a mean arterial blood pressure (MAP) of more than 65mmHg. If SVI falls by more than 25% in relation to the Starting-SVI (#) or SVI drops below 40ml/m² a fluid challenge with 200ml of a colloid solution is performed. The performance of a fluid challenge is not followed up to SVI is not increasing anymore as in published algorithm for preload optimization. Instead the volume administration is stopped if the SVI is higher than 40ml/m² and not lower than 25% of the Starting-SVI (#). If there is still central venous congestion measured by an increased central venous pressure (CVP) and the clinical judgment of venous bleeding during incision of the liver by the surgeon administration of either enoximone or nitroglycerine could be performed (^X^). After sealing the liver resection area volume administration can once again be guided to optimize SVI according to the previously published algorithms up to the end of surgery[20].

Haemodynamic management in the ODM and PPA guided group was performed according to a goal-directed algorithm adapted for liver surgery (Fig 2). This goal-directed algorithm was developed on the basis of an outcome-based goal-directed haemodynamic algorithm [20] which was adjusted for the demands of liver surgery.

The goal-directed algorithm guides the administration of intravenous colloid solution to maintain preload, the titration of norepinephrine to maintain arterial blood pressure and if necessary the titration of enoximone or nitroglycerine to lower central venous pressure. The algorithm is divided in three different time periods: (1) the initial preload optimization, (2) the haemodynamic therapy up to finishing the liver resection, and (3) the preload re-optimization after sealing the liver resection area.

After induction of anaesthesia and establishing of the advanced haemodynamic monitoring the haemodynamic algorithm predetermines a fluid challenge with 200ml of a colloid solution to optimize stroke volume index (SVI). If SVI increases by more than 10% additional fluid challenges with an intravenous bolus of 200 ml were given until no further increase in stroke volume of ≥10% could be measured. The value of SVI after the last fluid challenge is defined as Starting-SVI.

After determining the Starting-SVI the second time period of the haemodynamic algorithm starts up to the point the liver resection area is surgically sealed. During that period arterial blood pressure is maintained by titration of continuous administration of norepinephrine to reach a systolic blood pressure (SBP) of more than 100mmHg and a mean arterial blood pressure (MAP) of more than 65mmHg. If SVI decreases by more than 25% in relation to the Starting-SVI or SVI drops below 40ml/m² a fluid challenge with 200ml of a colloid solution is performed. At this time, the administration of a fluid challenge is not followed until no further increase in stroke volume of ≥10% could be measured but instead the volume administration is stopped if the SVI increased more than 40ml/m² and not lower than 25% of the Starting-SVI. If there is still central venous congestion measured by an increased central venous pressure (CVP) and the clinical judgment of venous bleeding during incision of the liver by the surgeon administration of either enoximone or nitroglycerine could be performed. The indication of a positive inotrope would be the clinical estimation of the need to lower right ventricular afterload and to strengthen the myocardial performance. For the titration of nitroglycerine the indication is the clinical estimation that the patient was previously volume overloaded to decrease central venous pooling.

After sealing the liver resection area a preload re-optimization is performed again to optimize SVI according to the previously published algorithms up to the end of surgery [20].

During volume administration and haemodynamic data collection there were no changes in anaesthetic treatment, ventilator settings, dose of norepinephrine or inotropic drugs and patient positioning. Prior to every optimization period the Doppler probe was readjusted to ensure an ideal placement. Every 30 minutes zeroing of arterial and central venous pressure and a fast flush test were performed.

### Endpoints

The primary endpoint of this study was stroke volume before the intraoperative start of the liver resection. Secondary outcome measures were all determined with the study period of 8 postoperative days and included intra- and postoperative haemodynamic parameters, blood losses, the cumulative frequency of organ dysfunctions (cerebral, pulmonic, renal, abdominal, cardiovascular), infections, satisfaction of the patients (pain levels), (serious) adverse events and hospital length of stay and time treated in the intensive care unit.

### Statistical Analysis

Because of limited sample sizes and non-normal distributions of the observations, data were expressed as median [25%, 75% quartiles], or frequencies [%], respectively. For the same reason, differences between the regarded groups in terms of interesting clinical parameters were univariately tested by using non-parametric exact Mann-Whitney tests for independent groups or exact Wilcoxon tests for pairwise comparisons within the intervention groups, respectively. Frequencies were (univariately) tested by the exact Mantel-Haenszel test (ordered categories) or the exact Chi-square test. Changes in interesting clinical outcomes with respect to time were analysed using multivariate nonparametric analysis of longitudinal data in a two-factorial design (1^st^ (independent) factor: groups, 2^nd^ (dependent) factor: repetitions in time). Therefore, all the time points were simultaneously compared on the corresponding response curves.

The following hypotheses have been tested with those analyses: differences between groups (over time) [Group], systematic changes in time (over groups) [Time overall], Interactions between groups and time [Group x Time] as well as systematic changes in time separately for every group [Time course].

After global testing, post-hoc analyses were carried out to detect specific differences between groups at fixed times (Mann-Whitney tests) or within groups with respect to interesting pairs of time points (Wilcoxon tests). A two-tailed p-value < 0.05 was considered statistically significant. All tests have to be understood in the area of exploratory data analysis. Therefore, no adjustments for multiple testing have been made.

Bland-Altman analysis for repeated measurements per patient were used to assess the agreement between ODM and PPA in terms of absolute values of stroke volume during the course of surgery. The bias was defined as the mean of differences between the two methods. The differences were regressed on the average to check for a constant or non-constant bias and variance. A linear mixed model with random effects was used to adjust for patient times replicate and method times patient interaction, resulting in a common standard deviation (SD) to calculate the limits of agreement (LOA’s) with upper (mean bias+1.96SD, ULOA) and lower (mean bias-1.96SD, LLOA) limits [21]. The percentage error (PE) was calculated as [1.96×SD of the bias/(mean(SVI_ODM_ + SVI_PPA_)/2)] [22]. The limit for an acceptable PE was redefined according to the precision of the monitors [23]. The precision of the ODM was previously reported ranging from 4.7% to 8.5% [24–26]; because there is no data on the precision of the PPA, we set the precision of the PPA at 20% [22]. Implying the worst reported precision of the ODM (8.5%), the redefined PE was therefore determined at < 21.7%.

Trending between ODM and PPA was analysed using the polar plot method [27, 28]. Trending between the two methods is shown by the angle from the polar axis and the magnitude of the change in SVI by the distance from the origin. In calculating the polar statistics, negative changes were converted to positive changes by rotating through 180°; central zone data (10% changes) were excluded due to intrinsic random error of the measurement of the ODM and PPA. Subsequently the mean polar angle (or angular bias) and the radial limits of agreement (RLOA’s, radial sector that contains 95% of the data points) with an upper (R-ULOA) and lower (R-LLOA) limit were determined. Acceptable trending is generally defined as an angular bias < ±5° and RLOA’s lying within radial limits of ±30° (boundary limits) [28]. Similar to Bland-Altman analysis the boundary limits for acceptable trending were adapted according to the precision of both monitors from ±30° to ±21.7° around the angular bias. The angular concordance rate was calculated as percentage of data points lying within ±21.7° limits.

All numerical calculations were performed with IBM SPSS Statistics, Version 21, Copyright 1989, 2010 SPSS Inc., SAS, Version 9 1, Copyright by SAS Institute, Inc., Cary, NC, USA, and the R project for Statistical Computing, Version 3.0.1.

## Results

Demographic data, baseline characteristics, intraoperative parameters and postoperative outcome data were not different between the study populations, except that the patients in the PPA group were significantly younger than in the ODM group (Table 1).

**Table 1:** General patient characteristics.

Parameter	ODM group (n = 11)	PPA group (n = 10)	P value ODM vs. PPA
**Age** (years)	69(56;75)	52(41;65)	#0.02
**Female**, n (%)	5 (45.5)	5 (50.0)	$1.00
**Body Mass Index** (kg/m²)	25(23;27)	24(22;29)	#0.93
**Charlson comorbidity index**	6.0(5.0;9.0)	6.0(5.0;9.3)	#0.20
**Metabolic Equivalent of Task Activity (MET) score**	5(4;5)	5(4;5)	#1.00
**Chronic medications**			
Beta blocker, n (%)	5(50)	1(11.1)	§0.14
ACEI, n (%)	4(40)	2(22.2)	§0.63
Statines, n (%)	1(10)	1(11.1)	§0.85
Calcium receptor antagonist, n (%)	3(30)	1(11.1)	§0.58
Diuretics, n (%)	1(10)	1(11.1)	§1.00
Analgetics, n (%)	2(18.2)	0(0)	§0.48
Other medications, n (%)	6(60)	6(66.7)	§1.00
**American Society of Anaesthesiology (ASA)**			§0.51
ASA Physical Status I, n (%)	1(10)	1(10)	
ASA Physical Status II, n (%)	6(60)	3(30)	
ASA Physical Status III, n (%)	3(30)	6(60)	
**Performed surgical procedure**			§0.82
Segment resection	0(0)	1(10)	
Hemihepatectomy	6(54.5)	4(40)	
Extended / trisection hepatectomy	5(45.5)	5(50)	
**Duration of surgery** (hh:mm)	04:00(03:20;05:45)	04:57(04:17;05:43)	#0.29
**Duration of anaesthesia** (hh:mm)	05:09(04:53;07:19)	06:23(05:29;07:11)	#0.40
**Fentanyl** (mg)	0.40(0.30;0.65)	0.50(0.29;0.65)	#0.99
**Remifentanil administration**, n (%)	5 (45.5)	7 (70)	$0.39
**Remifentanil highest Rate** (μg/kg/min)	0(0;0.20)	0.20(0;0.28)	#0.21
**Ketanest administration**, n (%)	4 (36.4)	8 (80)	$0.08
**Administration of piritramide or morphine,** (mg morphine equivalents)	5.0(0;5.0)	2.8(0;5.0)	#0.30
**Clonidin** (μg)	0(0;0)	0(0;41)	#0.21
**Estimated intraoperative blood loss** (ml)	720(392;1300)	450(350;975)	#0.57
**Red packed cells** (units per patient)	1.0(0;2.0)	0(0;2.5)	#0.64
**Fresh frozen plasma** (units per patient)	2.0(0;6.0)	2.0(2.0;8.5)	#0.53
**Patients requiring transfusion during hospital stay**, n (%)	9(82)	8(80)	$1.00
**Patients with complications,** n (%)	6 (55)	9 (90)	$0.15
**Total number of complications per patient** (number) [29, 30]	1.0 (0; 2.0)	2.0 (1.0; 3.0)	#0.27
**Highest grade of complication** [29, 30]			§0.25
Clavien grade 0, n (%)	5 (46)	1 (10)	
Clavien grade I, n (%)	0 (0)	0 (0)	
Clavien grade II, n (%)	3 (27)	6 (60)	
Clavien grade IIIa, n (%)	2 (18)	2 (20)	
Clavien grade IIIb, n (%)	1 (9)	1 (10)	
**LOS in PACU or HDU** (dd/hh)	00:21(00:18;01:03)	00:23(00:20;03:13)	#0.25
**LOS hospital preoperative** (dd:hh)	03:00(01:00;08:00)	02:00(01:18;03:06)	#0.45
**LOS hospital postoperative** (dd:hh)	13:00(12:00;19:00)	13:00(09:18;22:12)	#0.91
**Hospital discharge within 30 days**, n (%)	10(90.9)	9(90)	$1.00

Data are shown as median (25%; 75%) quartiles or as number n of patients (%). p-values calculated for the ODM versus the PPA group using the exact Wilcoxon-Mann-Whitney test #, the exact Mantel-Haenszel test (ordered categories)

§ or the exact Chi-square test

$ as appropriate.

Abreviations: ACEI: angiotensin converting enzyme inhibitor, LOS: length of stay, PACU: postanaesthesia care unit, HDU: high dependency care unit.

SVI before start of liver resection was SVI_**ODM**_ 49 (37; 53) ml m^-2^ in the ODM and SVI_**PCA**_ 48 (41; 56) ml m^-2^ in the PPA group, respectively (p = 0.40). In the ODM and PPA group the patients were haemodynamically stable with respect to arterial blood pressure and heart rate but in the PPA group there was an increase of norepinephrine levels up to the end of liver incision and then a decline again (Table 2). In contrast to the ODM group, the central venous pressure dropped in the PPA group during the liver incision (Table 2).

**Table 2:** Intraoperative haemodynamic parameters of the study groups.

Parameter	Time point	ODM group (n = 11)	PPA group (n = 10)	Non-parametric analysis for longitudinal data ODM vs. PPA:
**Mean arterial pressure** (**MAP**; mmHg)	T1	68 (61;91)	74 (58;88)	Group: 0.46
	T2	82 (76;96)	78 (70;86)	Time overall: 0.19
	T3	75 (69;81)	74 (69;84)	Group x Time: 0.55
	T4	76 (70;81)	75 (69;82)	Time ODM: 0.10
				Time PPA: 0.93
**Heart rate** (**HR**; beats/min)	T1	59 (54;80)	71 (53;84)	Group: 0.62
	T2	67 (64;88)	72 (66;87)	Time overall: 0.04
	T3	73 (67;83)	79 (73;108)	Group x Time: 0.52
	T4	76 (67;87)	74 (65;94)	Time ODM: <0.01
				Time PPA: 0.12
**Norepinephrine** (μg kg^-1^min^-1^)	T1	0(0;0.10)	0.01(0;0.04)	Group: 0.25
	T2	0.01(0;0.10)	0.04(0.03;0.10)	Time overall: <0.01
	T3	0.06(0;0.25)	0.15(0.06;0.27) #1	Group x Time: 0.04
	T4	0(0;0)	0(0;0) #2 #3	Time ODM: <0.01
				Time PPA: <0.01
**Central venous pressure** (**CVP**; mmHg)	T1	5 (2;7)	7 (6;11)	Group: 0.15
	T2	3 (2;5)	2 (1;4) #1	Time overall: <0.01
	T3	2 (-1;5)	2 (0;3) #1	Group x Time: 0.74
	T4	6 (2;8)	4 (3;8)	Time ODM: 0.09
				Time PPA: <0.01
**Cardiac index** (**CI**_**ODM**_; l min^-1^m^-2^)	T1	2.9(2.2;3.6)	3.8(3.0;5.0)	Group: 0.03
	T2	3.1(2.4;4.0)	3.0(2.5;3.5)	Time overall: <0.01
	T3	3.4(2.5;3.9)	2.8(2.3;3.1) #1	Group x Time: 0.44
	T4	4.0(3.0;5.0)	4.0(3.0;4.0) #3	Time ODM: 0.02
				Time PPA: <0.01
**Cardiac index** (**CI**_**LIDCO**_; l min^-1^m^-2^)	T1	2.4(2.2;3.5)	3.6(2.9;4.9)	Group: 0.96
	T2	3.0(2.3;3.9)	3.3(2.8;4.2)	Time overall: 0.41
	T3	3.0(2.2;3.9)	3.9(3.0;4.7)	Group x Time: 0.37
	T4	3.0(2.5;4.5)	4.0(3.0;5.5)	Time ODM: 0.08
				Time PPA: 0.76
**Stroke volume variation** (**SVV**_**LIDCO**_; %)	T1	6(5;9)	7(5;12)	Group: 0.20
	T2	7(5;12)	12(11;14) *	Time overall: 0.56
	T3	11(7;15)	11(8;18)	Group x Time: 0.17
	T4	8(5;12)	12(7;13)	Time ODM: 0.31
				Time PPA: 0.04
**Flow time corrected** (**FTc**_**ODM**_; ms)	T1	361(331;373)	381(372;430) *	Group: 0.1
	T2	352(338;377)	318(308; 373)	Time overall: <0.01
	T3	332(302;386)	298(269;343) #1	Group x Time: 0.4
	T4	358(332;380)	345(307;377)	Time ODM: 0.3
				Time PPA: <0.01
**Peak velocity** (**PV**_**ODM**_; cm s^-1^)	T1	61(53;92)	99(62;127)	Group: 0.67
	T2	62(57;64)	78(70;109) *	Time overall: <0.01
	T3	64(58;97)	80(56;104)	Group x Time: 0.10
	T4	69(63;108)	104(71;125)	Time ODM: 0.02
				Time PPA: 0.16
**Crystalloid** (ml)	T1	300(200;600)	175(100;1000)	Group: 0.86
	T2	1475(800;1600) #1	1200(800;1525)	Time overall: <0.01
	T3	1700(913;2088) #1	1650(1075;1885) #1	Group x Time: 0.48
	T4	1800(1300;2500) #1	2100(1588;2488) #1	Time ODM: <0.01
				Time PPA: <0.01
**Colloid** (ml)	T1	0(0;0)	0(0;200)	Group: 0.40
	T2	600(200;1000) #1	525(350;650)	Time overall: <0.01
	T3	700(500;1350) #1	625(400;800) #1	Group x Time: 0.41
	T4	1700(950;2450) #1	1300(950;1600)#1 #2	Time ODM: <0.01
				Time PPA: <0.01
**Crystalloid + Colloid** (ml)	T1	450(200;600)	425(100;1025)	Group: 0.54
	T2	1900(1675;2250) #1	1675(1400;1975)	Time overall: <0.01
	T3	2425(1863;2825) #1	2200(1888;2493) #1	Group x Time: 0.49
	T4	3300(2750;4725)#1 #2	3075(2800;3638)#1 #2	Time ODM: <0.01
				Time PPA: <0.01
**Diuresis** (ml)	T1	0(0;100)	115(53;163)	Group: 0.66
	T2	390(80;940) #1	385(148;830)	Time overall: <0.01
	T3	385(78;1055) #1	390(195;890) #1	Group x Time: 0.40
	T4	515(328;1193) #1	590(240;938) #1	Time ODM: <0.01
				Time PPA: <0.01

Data are shown as median (25%; 75%) quartiles over the time after start of surgery for the time points: T1 = first measurement of advanced haemodynamic monitoring / start of operation; T2 = before liver resection; T3 = after liver resection; T4 = end of operation. Statistical significances: # for comparisons with respect to time points within the group (exact Wilcoxon tests);

^#1^: p<0.05 vs. T1,

^#2^: p<0.05 vs. T2,

^#3^: p<0.05 vs. T3 and

* for comparisons between the ODM and PPA group (Mann-Whitney tests) with p<0.05 at the time point ODM group vs. PPA group. The nonparametric analysis for longitudinal data of the parameters between the ODM and the PPA group are outlined with corresponding p values.

**Fig 3:**
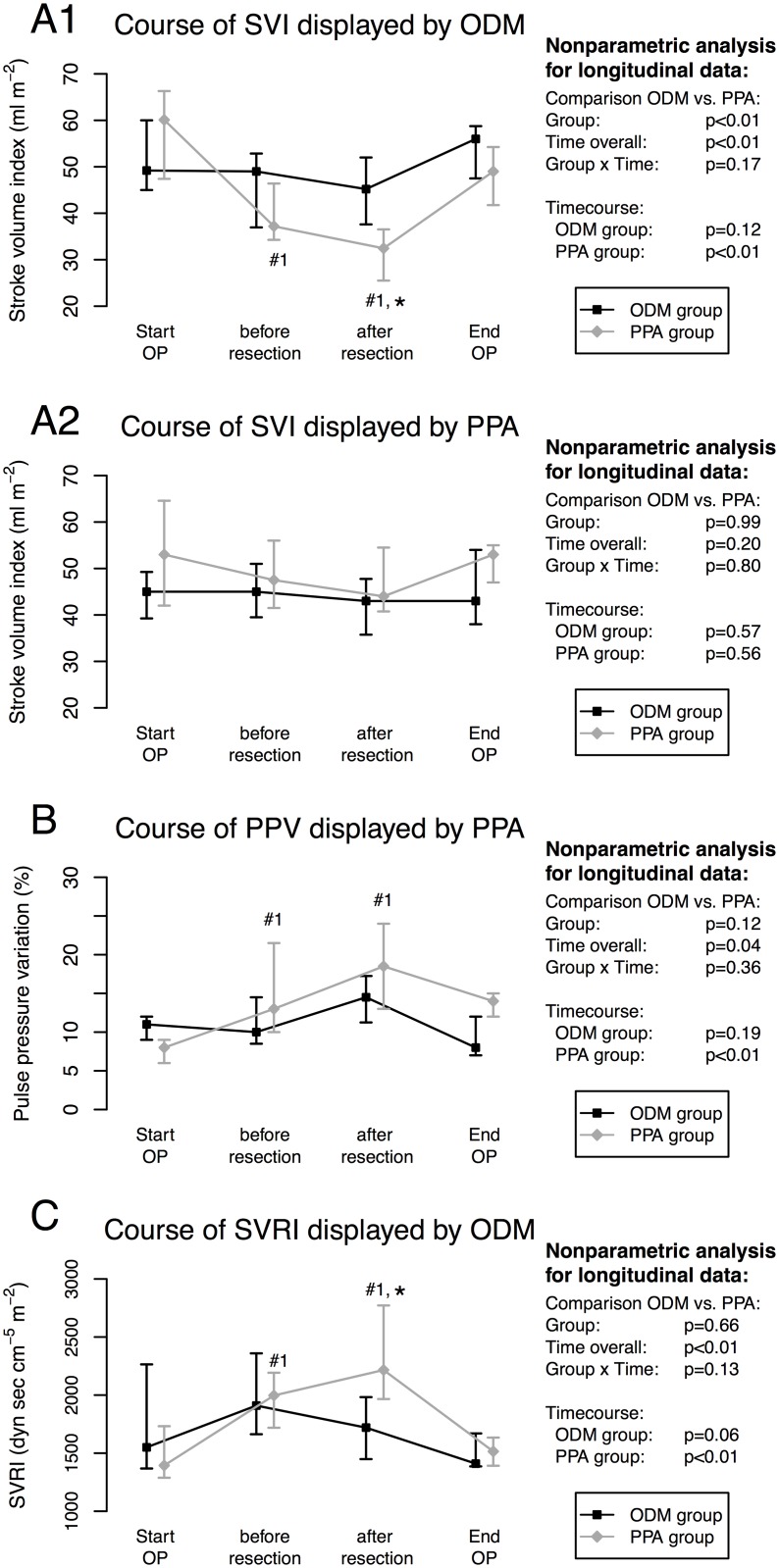
Time course of intraoperative haemodynamic parameters. Time course of stroke volume index (SVI) of the two intervention groups displayed by ODM (A1) and PPA (A2); time course of pulse pressure variation (PPV) of the two intervention groups displayed by PPA (B) and systemic vascular resistance index (SVRI) of the two intervention groups displayed by ODM (C). Data are shown as median (25%; 75%) quartiles over the time course of surgery and the nonparametric analysis for the parameters that are outlined with corresponding p values. Statistical significances: # for comparisons with respect to time points within the group (exact Wilcoxon tests); #1: p<0.05 vs. T1, #2: p<0.05 vs. T2, #3: p<0.05 vs. T3 and * for comparisons between the ODM and PPA group (Mann-Whitney tests) with p<0.05 at the time point ODM group vs. PPA group.

Patients of the ODM group had no decline of stroke volume index during the course of surgery measured by the oesophageal Doppler (p = 0.12) and the LiDCOrapid (p = 0.57). This was in contrast to the patients of the PPA group. They showed a decline of stroke volume index measured by the ODM over the time of surgery (p<0.01) while these haemodynamic changes were not reflected by the measurements of stroke volume index by the LiDCOrapid (p = 0.556) (Fig 3, 3A1 and 3A2). A drop of the corrected flow time (FTc) measured by the ODM (p<0.01), a higher stroke volume variability before start of liver incision, an increase of pulse pressure variability (p<0.01) in the PPA group and higher systemic vascular resistance index (SVRI) displayed by the ODM further suggest a haemodynamic deterioration throughout the course of surgery in the PPA group (Table 2, Fig 3B and 3C). Consequently, in the PPA group stroke volume index, cardiac index and FTc measured by the ODM showed a decrease and stroke volume variability (SVV) and PPV measured by the LiDCOrapid reflected an increase over the time course of the operation that was not found in the ODM group. Interestingly, these haemodynamic differences were not accompanied by differences of the cumulative administration of the crystalloid or colloid solutions or diuresis (Table 2).

**Fig 4:**
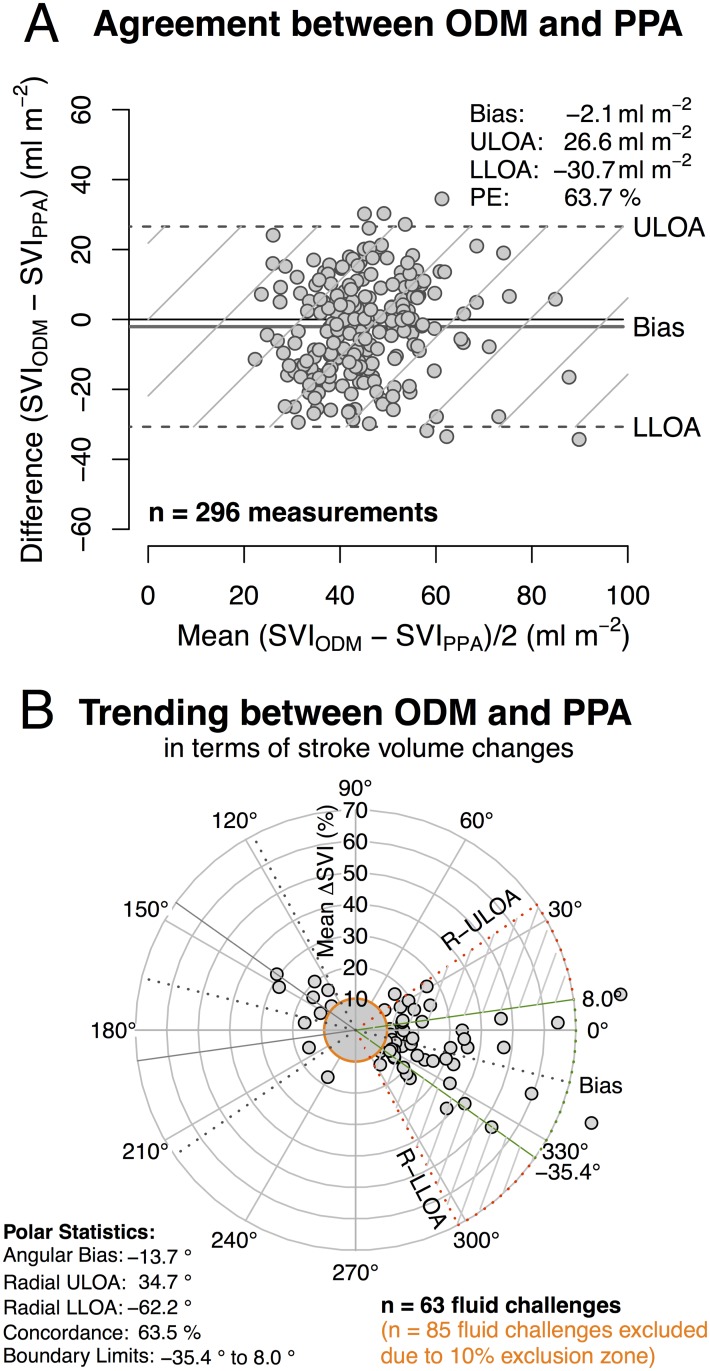
Bland-Altman and Polar Plot analysis. Bland-Altman plot for multiple measurements per patient assessing the agreement between ODM and PPA in terms of absolute values during the course of surgery (A) and Polar Plot analysis assessing trending of stroke volume during a fluid challenge between ODM and PPA (B). ULOA—Upper limit of agreement (bias+1.96SD); LLOA—Lower limit of agreement (bias-1.96SD); PE—percentage error; R-LLOA = Radial lower limit of agreement (bias-1.96SD). The shaded area (defined by RLOA’s and boundary limits) visualizes the magnitude of non-agreement between ODM and PPA.

A total of 148 fluid challenges in 21 patients (ODM and PPA group) were performed resulting in 296 paired measurements. In regard to absolute values Bland-Altman analysis revealed a constant bias and variance. ODM and PPA showed a poor agreement resulting in a percentage error of 63.7%. (Fig 4A) Analysing the percentage change of stroke volume during a fluid challenge Polar Plot analysis revealed a poor trending between ODM and PPA with an angular bias of -13.7°, radial limits of agreement of -62.2° to 34.7° and an angular concordance rate of 63.5% (Fig 4B).

**Fig 5:**
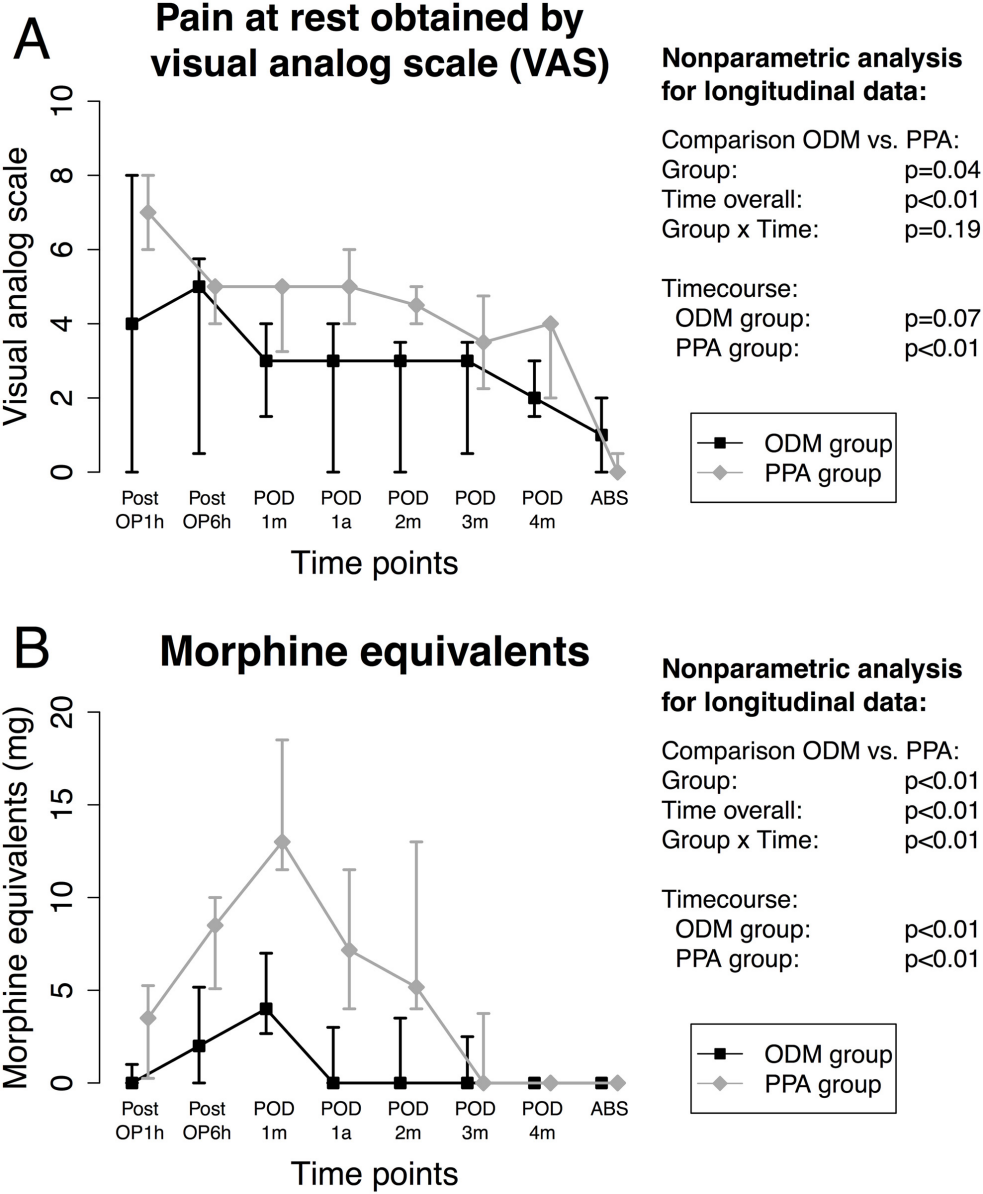
Postoperative pain and administration of morphine. Time course of postoperative pain at rest measured by the Visual Analog Scale (VAS) and the administered amounts of morphine equivalents (mg). Data are shown as median (25%; 75%) quartiles over the time after the end of surgery and the nonparametric analysis for the parameters that are outlined with corresponding p values.

Although there were no differences in intraoperatively administered opioids, ketanest or clonidine patients in the PPA group had higher pain levels even though receiving more morphine equivalents during the postoperative course (Fig 5).

The intraoperative use of the goal-directed haemodynamic algorithm adapted for liver surgery was feasible and showed high tolerance of the assigned anaesthetists. There were no surgical complaints of increased bleeding during liver incision and the anaesthetists followed the algorithm with a very high compliance. The administration of a positive inotrope or nitroglycerine as a safety criterion was not indicated by the study personnel or the assigned anaesthetist.

## Discussion

The main findings of the study are that (1) within a goal-directed algorithm adapted for liver surgery the ODM guided group was haemodynamically stable while within the PPA guided group advanced haemodynamic parameters suggested a potential hypovolaemia which was not indicated by the course of stroke volume measured by the PPA but reflected by simultaneous measurements of the ODM, (2) that the PPA guided group had significantly higher postoperative pain levels than the ODM group even though receiving more morphine equivalents during the postoperative course; and (3) that agreement and trending between ODM and PPA was poor.

To our knowledge this is the first study comparing the effect of the use of different haemodynamic monitors on the intraoperative haemodynamic parameters within a goal-directed haemodynamic algorithm in a blinded fashion. The primary endpoint of the study was stroke volume before liver incision. In regard to univariate analysis SVI at this time point was not significantly different between the ODM and the PPA group. However, regarding the time point after sealing the liver resection area SVI measured by the ODM was significantly higher in the ODM than in the PPA group but the measurements by the PPA were not different. We found the same results for the analysis of the time course during the entire course of the operation with a significant decline of SVI measured by the ODM in the PPA group but no differences in SVI measured by the PPA in the PPA group. As both monitors do not represent a gold standard for haemodynamic measurements it is not suitable to state solely by the measurements of stroke volume that patients were stable or unstable. However, further haemodynamic indicators showed that the patients in the PPA group although being younger had a significant deterioration of norepinephrine levels, a substantial drop of the FTc and an increase of SVV and PPV during the course of surgery suggesting a potential hypovolaemia. These indicators combined with the discrepancies in SVI measurements might indicate that haemodynamic optimization by the LiDCOrapid is not comparable to haemodynamic treatment based on the oesophageal Doppler. This fact could also be clarified by the clinical poor agreement and trending between ODM and PPA. The poor agreement of the absolute values of stroke volume index between ODM and PPA is consistent with the results of a recent study evaluating these monitors in colorectal surgery [31]. The poor trending of stroke volume index between the ODM and the PPA seems to be the substantial issue as therapeutic decisions are based on the ability to test and confirm patient’s haemodynamic response to appropriately guide haemodynamic therapy and it supports the fact that haemodynamic therapy based on these monitors might be different but as none of the monitors represents a gold standard for haemodynamic measurements conclusions of this fact have to be drawn very carefully.

A low CVP of 2 to 5 or below 5mmHg as a target for haemodynamic management in liver surgery reduces intraoperative blood losses [4–6] and improves postoperative outcome with a reduced hospital length of stay [5–7]. Due to a higher CVP at the beginning of the operation there was a significant reduction of the CVP in the PPA and not in the ODM group. However, in both groups the targeted low CVP was effectively reached by using the algorithm. In the ODM group a low CVP was reached without a drop of SVI, CI or FTc or an increase in norepinephrine levels, SVV or PPV. This indicates that the haemodynamic management within a goal-directed haemodynamic algorithm guided by oesophageal Doppler was able to reduce backflow in the liver while maintaining circulatory flow. As this study was not planned to evaluate the clinical impact of the use of advanced haemodynamic monitors further studies are needed to direct this question.

The differences in postoperative pain levels and administrated morphine equivalents could be an indicator of clinical association of an intraoperative deteriorated haemodynamic status and the postoperative consequence of increased pain. In acute bleeding in healthy volunteers [11] a drop of stroke volume was associated with a deteriorated gastric microperfusion and in chronic heart failure one of the first areas with a reduced perfusion was the upper gastro-intestinal (GI) region [32]. That is why we think that a reduced blood flow in the surgical area of the study patients could be a contributor to the development of postoperative pain in upper GI surgery. This is the first study to our knowledge to show the association of a deviated haemodynamic status to postoperative pain and we think this should lead to the point that in future haemodynamic studies pain should be a secondary outcome measure. However, we cannot exclude that the differences in pain are associated to the differences of ages between study groups, but to our knowledge there is no study published showing this relationship.

The adapted goal-directed haemodynamic algorithm to guide volume and catecholamine administration in liver surgery was feasible and showed a high clinical tolerance. After primary preload optimization the algorithm could prevent substantial haemodynamic instability reflected by the current used open monitor up to the sealing of the liver resection area without any complaints of increased bleeding tendency during liver incision. To our knowledge this is the first algorithm to guide volume and catecholamine administration by advanced haemodynamic monitoring measuring circulatory flow in liver surgery.

Goal-directed optimization by the oesophageal Doppler and the LiDCOrapid showed differences in intraoperative cardiovascular parameters, postoperative pain and opioid administration. Again, it is not possible to state that one monitor might be wrong and another is better but it can be stated out of our data that haemodynamic optimization with the oesophageal Doppler is not the same as with the LiDCOrapid and vice versa. As the recommendations to perform goal-directed haemodynamic therapy is grossly based on studies using the oesophageal Doppler it seems important to us that based on the results presented here the use of the LiDCOrapid for goal-directed haemodynamic therapy has to be confirmed in further prospective randomised controlled studies to confirm its beneficial clinical impact during anaesthesia.

## Supporting Information

S1 CONSORT Checklist CONSORT Checklist. (DOC)

S1 Doc Description of the clinical perioperative pathway of the study patients. (DOC)

S1 Fig Polar Plot analysis of changes of stroke volume and mean arterial pressure. Polar Plot analysis assessing trending of stroke volume during a fluid challenge measured by ODM (A) and PPA (B) and change of mean arterial pressure during the fluid challenge (MAP). ULOA—Upper limit of agreement (bias+1.96SD); LLOA—Lower limit of agreement (bias-1.96SD); PE—percentage error; R-LLOA = Radial lower limit of agreement (bias-1.96SD). The shaded area (defined by RLOA’s and boundary limits) visualizes the magnitude of non-agreement between ODM (A) and PPA (B) and MAP. *The Angular concordance rate was significantly different between ODM-MAP and PPA-MAP trending (P value = 0.021).
(TIFF)

S1 Protocol Protocol Ethical application German. (DOC)

S2 Protocol Protocol Ethical application English. (DOC)
