# Supplementary material for: Haemodynamic Optimization by Oesophageal Doppler and Pulse Power Wave Analysis in Liver Surgery: A Randomised Controlled Trial
Source: PLoS One. 2015 Jul 17;10(7):e0132715. doi: 10.1371/journal.pone.0132715 (PMC4505861; doi:10.1371/journal.pone.0132715)
Supplement: S1 Doc — (DOC) [file pone.0132715.s002.doc]

**Appendix to the Submission:**

**Title**

**Haemodynamic Optimization by Oesophageal Doppler and Pulse Power Wave Analysis in Liver Surgery: A Randomised Controlled Trial**

**Running head:**

Haemodynamic Optimization by ODM and PPA in Liver Surgery.

**Methods**

**Clinical pathway:**

Briefly, after the pre-anaesthesiological visit the day before surgery the patients were allowed to drink clear liquids up to two hours and ingest solid food up to six hours prior to anaesthesia. Thirty minutes before being called to the operation area the patients received midazolam 3.75 to 7.5mg p. o. After establishing an intravenous line and basic monitoring (electrocardiogram, oxygen saturation and oscillotonometric arterial blood pressure measurement) induction of anaesthesia was performed with thiopental (0.6mg kg BW) or propofol (2mg kg BW) and fentanyl (1 to 3µg kg BW). Maintenance of anaesthesia was conducted by desflurane (0.7 to 1.2 MAC) or propofol (6 to 10mg kg BW) to maintain BIS values between 40 and 55 and pain block was reached by bolus administration of fentanyl and supplemental continuous remifentanil and bolus administration of ketamine and piritramide for postoperative pain according to the clinical estimation of the treating anaesthetist. After induction of anaesthesia a central venous catheter in the right internal jugular vein, an arterial line in the radial artery and a urine catheter were placed under sterile conditions. The oesophageal Doppler (CardioQ^TM^, Deltex Medical, Chichester, Great Britain) was placed and the pulse power analysis (LiDCOrapid^TM^, LiDCO Ltd., London, Great Britain) was connected to the arterial line module of the intellivue monitor (Intellivue monitor, Philips GmbH, Hamburg, Germany). During surgery patients were ventilated pressure-controlled with a tidal volume of 8ml kg BW, a positive-end-exspiratory-pressure of 5mmHg and respiratory rate was adapted to maintain end-tidal CO_2_ between 35 and 42mmHg. Frequent arterial blood gas analysis was performed to confirm normoventilation. Blood losses were substituted guided to the haemoglobin values in combination with the incidence of acute bleeding transfusions with red-packed-cells according to the actual transfusion guidelines. The administration of fresh-frozen-plasma products were performed if there were clinical signs of a bleeding tendency or in haemodynamically unstable patients according to the clinical estimation of the treating anaesthetists. Surgically the operations were performed following clinical standards of the Department of General, Visceral, and Transplantation Surgery. Postoperatively the patients were extubated and transferred to an intermediate or intensive care unit. The patients were discharged from the IMCU / ICU if defined criteria were fulfilled being cardiopulmonary and metabolically stable without signs of renal or gastrointestinal failure or present infection or not sufficiently controlled pain. The fluid and volume therapy and catecholamine administration was performed according to the clinical estimation of the treating physicians. Treatment of pain was directed by the administration of metamizol and piritramide according the pain levels expressed by the patients according to the numeric rating scale (NRS) and their wish to receive pain medications. The study team visited the study patients daily for data acquisition and screening for adverse events up to the 8^th^ postoperative day and before hospital discharge but was not involved in postoperative treatment of any kind.
