# Supplementary figures and images for: Haemodynamic Optimization by Oesophageal Doppler and Pulse Power Wave Analysis in Liver Surgery: A Randomised Controlled Trial
Source: PLoS One. 2015 Jul 17;10(7):e0132715. doi: 10.1371/journal.pone.0132715 (PMC4505861; doi:10.1371/journal.pone.0132715)

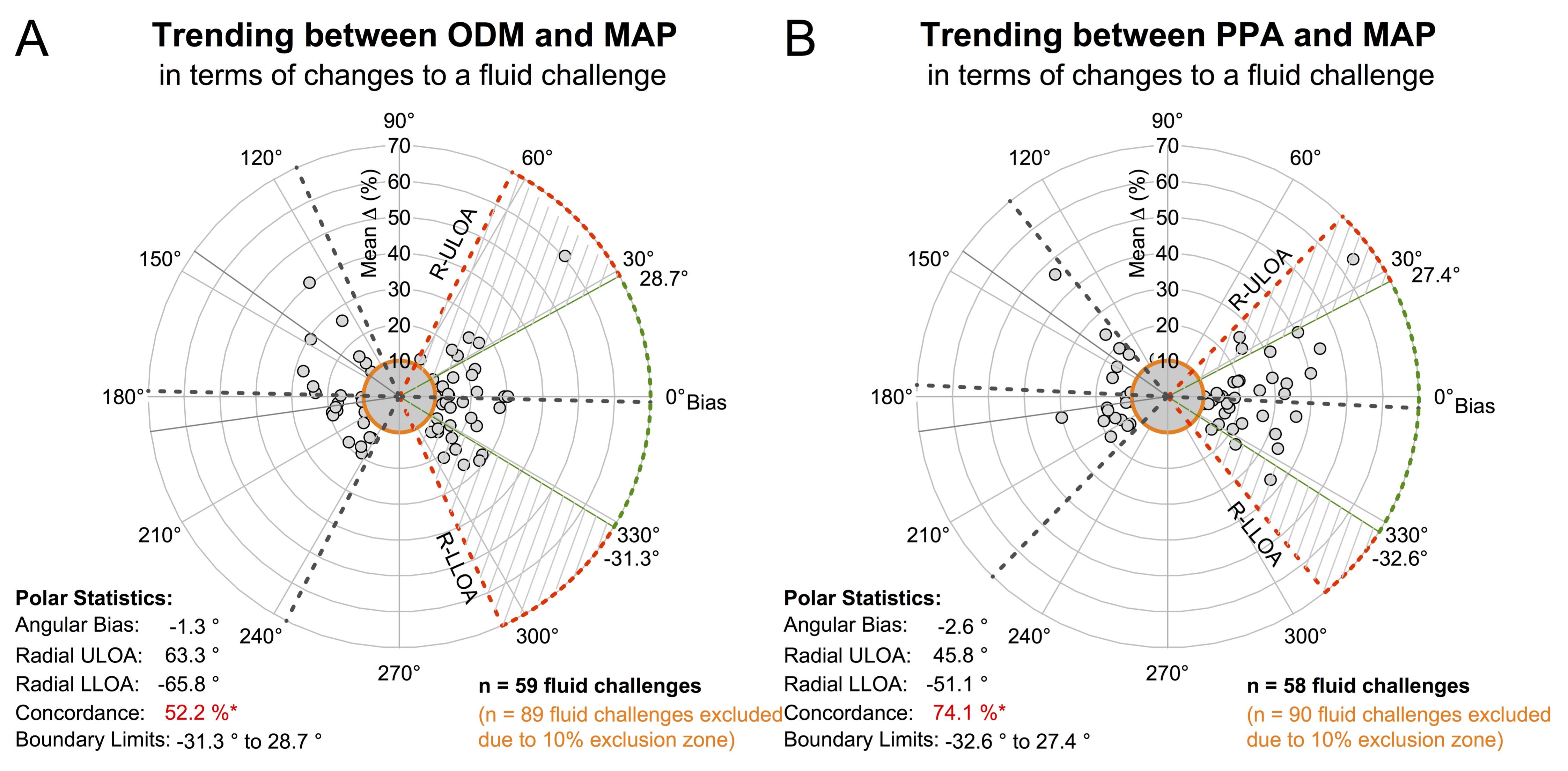

Supplement: S1 Fig — Polar Plot analysis assessing trending of stroke volume during a fluid challenge measured by ODM (A) and PPA (B) and change of mean arterial pressure during the fluid challenge (MAP). ULOA—Upper limit of agreement (bias+1.96SD); LLOA—Lower limit of agreement (bias-1.96SD); PE—percentage error; R-LLOA = Radial lower limit of agreement (bias-1.96SD). The shaded area (defined by RLOA’s and boundary limits) visualizes the magnitude of non-agreement between ODM (A) and PPA (B) and MAP. *The Angular concordance rate was significantly different between ODM-MAP and PPA-MAP trending (P value = 0.021). (TIFF) [file pone.0132715.s003.tiff]
