# Supplementary material for: Haemodynamic Optimization by Oesophageal Doppler and Pulse Power Wave Analysis in Liver Surgery: A Randomised Controlled Trial
Source: PLoS One. 2015 Jul 17;10(7):e0132715. doi: 10.1371/journal.pone.0132715 (PMC4505861; doi:10.1371/journal.pone.0132715)
Supplement: S1 Protocol — (DOC) [file pone.0132715.s004.doc]

Antrag auf Beratung durch die Ethikkommission zur Durchführung eines medizinisch-wissenschaftlichen Vorhabens, welches nicht die klinische Prüfung eines Arzneimittels beinhaltet.

| 1. a Titel der Studie | Der intraoperative Vergleich eines zielorientierten Hämodynamikprotokolls mittels des Monitorings mit dem LiDCOrapid oder dem ösophagealem Doppler mit konventioneller Therapie bei Leberteilrektionen – Eine Pilotstudie. |
| --- | --- |
| 1. b Kurztitel der Studie | Hämodynamik bei Leberresektionen |
| 2. Ethikkommissions -Antragsnummer | Wird von der Ethik-Kommission vergeben |
| 3. Entscheidungen anderer Ethikkommissionen in derselben Sache | Entfällt |
| 4. Gegenstand der Studie und ihre Ziele (Hypothesen, getrennt in Haupt- und Sekundärhypothesen) | Haupthypothese:  Die Verwendung eines intraoperativen, zielorientierten Hämodynamikprotokolls mittels des Monitorings mit dem LiDCOrapid oder den ösophagealem Doppler verbessert die Hämodynamik gemessen an dem Schlagvolumen des Herzens im Vergleich zur Standardtherapie bei Leberteilresektionen.  Sekundärhypothese 1:  Die Verwendung eines intraoperativen, zielorientierten Hämodynamikprotokolls mittels des Monitorings mit dem LiDCOrapid oder dem ösophagealem Doppler vermindert im Vergleich zur Standardtherapie das Auftreten perioperativer Dysfunktion (zerebral, pulmonal, renal, abdominal und kardiovaskulär), den intraoperativen Blutverlust und die postoperative Inzidenz von Infektionen und Lebensqualität bei Leberteilresektionen.  Sekundärhypothese 2:  Die Verwendung eines intraoperativen, zielorientierten Hämodynamikprotokolls mittels des Monitorings mit dem LiDCOrapid oder den ösophagealem Doppler verbessert im Vergleich zur Standardtherapie die postoperative Leberfunktion bei Leberteilresektionen.  Studiengruppen:  Die Steuerung der Volumen- und Katecholamin-therapie erfolgt gemäß:   1. Konventioneller Standardtherapie 2. Zielorientiertem Hämodynamikprotokoll basierend auf dem ösophagealem Doppler 3. Zielorientiertem Hämodynamikprotokoll basierend auf dem LiDCOrapid-Monitors   Primäre Zielgröße:  Das Schlagvolumen des Herzens vor dem intraoperativen Leber-Resektionsbeginn.  Sekundäre Zielgrößen:   - Hämodynamische Parameter intra- und postoperativ - Blutverlust intra- und postoperativ - Kumulative Anzahl an Organdysfunktionen (zerebral, pulmonal, renal, abdominal und kardiovaskulär) - Postoperative Leberfunktionstestung mit der LIMAX-Testung, ICG-Clearance, Lebergefäßfluss im Doppler und enzymatische und laborchemische Parameter - Körpergewichtsverlauf perioperativ - Postoperative Inzidenz von Infektionen - Zufriedenheit des Patienten, des Chirurgen und des Anästhesisten - Dauer bis zur Erfüllung der Krankenhausentlassungskriterien - Krankenhaus- und Intensivstationsverweildauer - Lebensqualität (gemessen am EQ-5D) - Verlauf endothelialer und immunologischer Parameter im perioperativen Verlauf |
| 5. Erläuterung der Bedeutung der Studie | Das hämodynamische Management von Leberteilresektion durch die Anästhesiologie ist bestimmt durch das Ziel die intraoperative Blutung im Rahmen der chirurgischen Durchschneidung der Leber zu minimieren ^1^. Dieses Ziel wird durch ein sehr hypovolämes Volumen- und Flüssigkeitsregime zur Erzielung eines niedrigen zentral-venösen Druckes verfolgt. Dadurch wird der Rückstau des Blutes vom rechten Herzen über die Lebervenen zur Resektionsfläche minimiert und der Blutverlust wird vermindert. Dieses Vorgehen ist durch einige Studien aus den 90er Jahren als Möglichkeit zur Reduktion der perioperativen Morbidität und Mortalität belegt ^2-4^.  In den letzten Jahren hat sich aber das Patientengut insofern verändert, als dass die Patienten häufiger mit Komorbiditäten vor allem aus dem kardiovaskulären Bereich zur Operation vorgestellt werden. Daraus ergibt sich aus unserer klinischen Erfahrung das Problem, dass einige Patienten durch das sehr restriktive Flüssigkeits- und Volumenmanagement einen sehr starken Abfall des Schlagvolumens und des Herzindex mit konsekutiver Minderperfusion vor allem der Splanchnikusregion aufweisen.  Mit dem ösophagealem Doppler und dem LiDCOrapid stehen heute zwei minimal-invasive Verfahren zur Verfügung, um das Schlagvolumen des Herzens sicher zu messen und damit die Steuerung der Volumen- und Katecholamintherapie ermöglichen zu können. Für kolorektale und gynäkologisch-urologische Resektionen und in der Traumatologie ist der Vorteil des erweiterten Monitorings auf den Krankenhausverlauf der Patienten gut durch Studien belegt (Auflistung im Anhang 1). Diese Studien zielen aber auf eine intraoperative Vorlastoptimierung im Sinne des Frank-Starling-Mechanismus. Für Leberteilresektion ist dieses Vorgehen aufgrund des damit verbunden erhöhten Risikos intraoperativer Blutungen nicht möglich. Daher ist von uns ein entsprechender Algorithmus für ein restriktives Volumenmanagement erstellt worden, um den Herzindex und die Splanchnikusperfusion ohne Vorlasterhöhung im Verlauf der Operation zu optimieren. Es gibt unseres Wissens nach bisher keinen Algorithmus in der Literatur zu einem zielorientiertem, restriktiven Volumenmanagement bei Leberteilresektionen.  Das Ziel dieser Studie ist es, die in unserer Klinik etablierten Protokolle eines intraoperativen, restriktiven, zielorientierten Volumenprotokolls mittels erweiterten hämodynamischen Monitorings gegen die Standardversorgung zu vergleichen, um den Einfluss auf das Schlagvolumen des Herzens als primären Parameter und auf die Beeinflussung des chirurgischen Vorgehens und der postoperativen Verlaufsparameter als sekundäre Ziele zu untersuchen.  Ein weiteres Ziel ist es im Rahmen der Studie interdisziplinär in Zusammenarbeit mit den Kollegen der Klinik für Klinik für Allgemein-, Visceral- und Transplantationschirurgie die einzelnen Module perioperativer Versorgung zu beschreiben und durch deren Standardisierung den Ergebnissen der Studie eine höhere Qualität und Transparenz zu verschaffen. |
| 6. Welche der folgenden Bestimmungen finden Anwendung   1. Medizinproduktegesetz -gemäß § 20 MPG (Gerät besitzt nicht die Konformitätserklärung oder diese liegt vor und es wird eine andere Indikation geprüft oder es werden zusätzlich invasive oder andere belastende Untersuchungen durchgeführt) **oder** -gemäß § 23MPG ? 2. Strahlenschutzverordnung § 23 3. Röntgenverordnung § 28 a 4. Gentechnikgesetz 5. Datenschutzgesetze | Datenschutzgesetze |
| 7. Ggf.: Bezeichnung und Charakterisierung der Prüfprodukte (z.B. Geräte bei MPG-Studien; bitte Anlagen beifügen) | Entfällt |
| 8. Wesentliche Ergebnisse der vorklinischen Tests oder Gründe für die Nichtdurchführung derselben | Die Methode des minimal-invasiven Monitorings der Herzkreislaufparameter ist hinreichend am Menschen evaluiert. Diese Studien werden im Anhang 1 und 2 und unter Punkt 9 aufgeführt. Aufgrund dessen wird auf die Darlegung vorklinischer Studien verzichtet. |
| 9. Wesentlicher Inhalt und Ergebnisse der vorangegangenen Studien/Anwendungen der in der Studie zu prüfenden Produkte | Der ösophageale Doppler wird seit etwa 15 Jahren in klinischen Studien verwendet. Eine Auswahl der Studien ist im Anhang 1 dargestellt. Der ösophageale Doppler in Verbindung mit einem auf den gemessenen Werten basierenden Volumenprotokoll konnte positive Effekte in der kardiovaskulären, urologisch-gynäkologischen, abdominalchirurgischen und traumatologischen Chirurgie nachweisen.  Durchgeführte Vergleiche mit anderen invasiven Methoden zur Bestimmung des Herzminutenvolumens zeigte eine gute Korrelation der Messergebnisse zwischen den Methoden.  Die Methode des erweiterten hämodynamischen Monitorings durch den LiDCOrapid ist sowohl für die Intensivstation als auch für die intraoperative Verwendung gegen andere etablierte Methoden getestet worden (dargestellt im Anhang 2). Dabei zeigte sich eine gute Korrelation bei verschiedenen hämodynamischen Situationen gegenüber den Standardmethoden. Im Gegensatz zum ösophagealen Doppler sind aber bisher keine Studien zur Untersuchung eines LiDCOrapid-basierten Algorithmus auf den Krankenhausverlauf publiziert worden.  Die Messung der Leberfunktion soll in dieser Studie über die weit verbreitete ICG-Clearance sowie den neu entwickelten LIMAX-Test durchgeführt werden. Dieses Verfahren beruht auf der kinetischen Analyse nach intravenöser Bolusinjektion von 13C-Methacetin (2mg/kg KG). Der einzige geschwindigkeits-bestimmende Schritt ist die Demethylierung durch das CYP1A2 in der Leber. Das entstehende 13CO2 wird in der Ausatemluft online mit der nichtdispersiven isotopenselektiven Infrarotspektroskopie (NDIRS) direkt am Patientenbett gemessen und gibt somit einen direkten Wert für die metabolische Leistung der Leber ^5^. Beide Verfahren stellen in der Klinik für Allgemein-, Visceral- und Transplantationschirurgie der Charité – Universitätsmedizin Berlin, Campus Virchow-Klinikum Standardverfahren zur Erfassung der prä- und postoperativen Evaluation von Patienten zur Leberteilresektion dar und werden im klinischen Verlauf zur Sonographie der Lebergefäße und den MRT-Untersuchungen der Leber korreliert. |
| 10. Beschreibung der vorgesehenen Maßnahmen/Untersuchungsmethoden und eventuelle Abweichungen von den in der med. Praxis üblichen Maßnahmen/Untersuchungen (was ist „Routine“, was wird davon abweichend in der Studie gemacht?) | Die perioperative Behandlung erfolgt für alle Patienten nach den publizierten und zertifizierten (nach DIN EN ISO 9001) Standards der Universitätsklinik für Anästhesiologie mit Schwerpunkt operative Intensivmedizin.  Es erfolgt bis auf den zusätzlichen Einsatz der nicht-invasiven Messung der Gewebeoxygenierung und des transkraniellen Dopplers keine studienbedingte Anpassung der Maßnahmen oder der Untersuchungen. Das beinhaltet die Narkosevorbereitung, die Induktion und Aufrechterhaltung sowie die postoperative Überwachung im Aufwachraum bzw. auf den Intensivpflegestationen und das indizierte invasive Monitoring der Patienten. Die Untersuchungen des Blutes des Patienten entsprechen ebenfalls der Routine-Diagnostik der beteiligten Kliniken. Die Werte der Blutuntersuchungen werden in Zusammenarbeit mit Dr. Weimann des Zentralinstitut für Laboratoriumsmedizin und Pathobiochemie am Campus Virchow-Klinikum hingehend wissenschaftlicher Fragestellungen bearbeitet, ohne dass sich daraus eine zusätzliche Blutabnahme für den Patienten ergäbe. Davon unabhängig werden den Patienten im Rahmen der klinischen Routine der Klinik für Allgemein-, Visceral- und Transplantationschirurgie Laborparameter zur Bestimmung der Leberregeneration und der Leberfunktion abgenommen. Aus rein wissenschaftlicher Sicht erfolgen im klinischen Behandlungsablauf hingegen nur die Blutabnahmen zur Bestimmung der perioperativen immunologischen und endothelialen Funktion. Die Entlassung aus dem Aufwachraum bzw. den Intensivpflegestationen wird aufgrund der Erfüllung der Entlassungskriterien nach dem Aldrete Score festgelegt (siehe Anhang 9 und 10).  Nach der Aufnahme auf den Stationen der Klinik für Allgemein-, Visceral- und Transplantationschirurgie werden die Routine-Aufnahme-Untersuchungen des klinischen Behandlungspfades für Leberteilresektionen durchgeführt. Es werden die Patienten auf Ein- und Ausschlusskriterien gescreent und nach Einschluss werden die Fragebögen zur Lebensqualität (EQ-5D, siehe Anhang 8, ebenso bei Studienabschluss), der CAGE- bzw. AUDIT-Test zur Erfassung von Alkoholerkrankungen mit den Patienten ausgefüllt. Die Patienten werden angehalten und motiviert orale Flüssigkeiten bis zwei Stunden vor dem Narkosebeginn zu sich zu nehmen.  Die Einleitung der Narkose erfolgt lediglich unter einer Volumengabe von unter 200 ml einer balancierten kristalloiden Lösung zur Sicherstellung der Applikation der Medikamente, die über den intravenösen Zugang gegeben werden sollen.  Zur Erfassung von Blutdruckabfällen im Rahmen der Narkoseeinleitung wird der Blutdruck minütlich bis zur Etablierung des ösophagealen Dopplers gemessen. Die nicht-invasiven Messungen der Gewebeoxigenierung und der kranialen Doppler-Flüsse erfolgt während der Operation. Bei kurzzeitig auftretenden Hypotensionen ist die Gabe von Effortil 2 mg i.v. und bei dauerhafter Hypotension die kontinuierliche Gabe von Noradrenalin zur Aufrechterhaltung der Ziel-Blutdrücke zu indizieren.  Nach Lagerung des Patienten im Saal und etablierter Doppler-Messung wird in der Doppler-Gruppe das Volumenprotokoll (siehe Anhang 3) mittels der Werte des erweiterten hämodynamischen Monitorings begonnen. In der ersten Gruppe besteht das Monitoring in der Messung der Werte durch den ösophagealen Doppler, in der zweiten in der Messung der Werte mit dem LiDCOrapid-Monitor. Dabei erfolgt in beiden Protokoll-Gruppen eine initiale Volumen-Gabe von 200 ml Gelatine-Lösung. Wenn der Patient Zeichen der Volumenreagibilität durch Anstieg des Schlagvolumens zeigt, wird die Volumen-Gabe solange wiederholt, bis das Schlagvolumen nicht weiter ansteigt. Anschließend erfolgen die Einstellung des Blutdrucks mit den Ziel-Werten nach den präoperativen Blutdruckwerten der Patienten (siehe unten) und die Aufrechterhaltung eines Herzindex über 2,5 l/min bis zum Resektionsende. Nach Beendigung der Maßnahmen an der Leber wird eine erneute Optimierung der Vorlast durch Volumen-Gaben unter Kontrolle des Schlagvolumens erzielt.  In der Kontroll-Gruppe erfolgt ebenso die Messung der Kreislaufverhältnisse mittels des LiDCOrapid verblindet gegen die anwesenden behandelnden Ärzte.  In der Kontroll-Gruppe erfolgt die Volumentherapie nach den Standards der Klinik für Anästhesiologie und operative Intensivmedizin. Dazu erfolgt die Bewertung des Volumenbedarfs unter restriktiven Gesichtspunkten anhand der erfassbaren Parameter wie Blutdruck, Herzfrequenz, Urinproduktion, periphere Durchblutung und abgeleiteten Parametern einer invasiven Blutdruckmessung, wenn sie bei dem Patienten aus Gründen von relevanten Vorerkrankungen indiziert war. In der Kontroll-Gruppe kann der Anästhesist nach Erfahrungswerten die Gabe von Vasopressoren oder positiv inotroper Medikamente indizieren. Das Ziel der Volumen- und Kreislauftherapie muss so gewählt sein, dass sowohl der Blutdruck entsprechend dem Zielblutdruck der präoperativen Messungen angepasst wird als auch ein normfrequenter Rhythmus (Herzfrequenz zwischen 40 und 100 Schlägen/Minute) angestrebt wird.  Die Patienten sollen eine intraoperative Volumentherapie mit balancierten Kristalloiden (z.B. Jonosteril ®) und unbalancierten Kolloiden (z.B. Gelafundin ®) erhalten.  Definition des Ziel-Blutdruck (mittlerer arterieller Blutdruck) aus den präoperative Werten:  - Normotensive Patienten: > 70 mmHg  - Hypertensive Patienten: > 80 mmHg  - Hypotensive Patienten: > 60 mmHg  Die Operation erfolgt nach den üblichen klinischen Richtlinien der Klinik für Allgemein-, Visceral- und Transplantationschirurgie.  Jeder Patient wird am Operationstag auf einer Intensivüberwachung bis zur Erfüllung der Entlassungskriterien aufgenommen. Direkt postoperativ werden die Routine-Laboruntersuchungen abgenommen. Der Patient kann direkt postoperativ, wenn der Patient ausreichend wach ist, gute Schutzreflexe hat und keine Übelkeit oder Erbrechen aufweist und keine chirurgische Kontraindikation vorliegt, mit dem Kostaufbau mit Flüssigkeit ad libitum und nachfolgend Joghurt und nach zweistündiger Toleranz feste Kost nach Wahl beginnen.  Die Volumen- und Flüssigkeitstherapie der Patienten im postoperativen Verlauf erfolgt entsprechend den Maßgaben der beteiligten Kliniken und der Erfahrungen der behandelnden Ärzte. |
| 11. Bewertung und Abwägung der vorhersehbaren Risiken und Nachteile der Studienteilnahme gegenüber dem erwarteten Nutzen für die Studienteilnehmer und zukünftig erkrankte Personen (Nutzen-Risiko-Abwägung) | Die Risiken der Verwendung der ösophagealen Doppler-Sonde sind als minimal zu bewerten. Die Sonde ist laut Deltex-Medical bisher etwa 800.000 Mal verwendet worden und dabei ergab sich nur einmal ein Verdacht auf eine ösophageale Verletzung. Die bisherigen Studien geben keine Beobachtungen von Doppler-assoziierten Komplikationen an. Auf eine nasale Anlage der Sonde mit dem eventuellen Risiko einer nasalen Blutung wird verzichtet. Die Sonden werden in dieser Studie oral platziert werden.  Den minimalen Risiken stehen die Daten der bisherigen klinischen Studien gegenüber, in denen mittels des ösophagealen Dopplers die Rate an Komplikationen, die Intensivstations- und Krankenhausverweildauer reduziert werden konnte.  Für den LiDCOrapid besteht für die Patienten kein zusätzliches Risiko, da der Monitor lediglich die Werte der invasiven Blutdruckmessung ableitet und über Berechnungsalgorithmen die Puls-Power der Herzschläge aus der Blutdruckkurve und nachfolgend das Schlagvolumen errechnen kann. Da jeder Patient aufgrund der Operation einen arteriellen Zugang erhält, besteht für die Patienten keine zusätzliche Gefahr.  Die Entwicklung und Evaluation in klinischer Hinsicht eines hämodynamischen Algorithmus für Leberteilresektionen auf der Basis minimal-invasivem Monitorings steht im günstigen Verhältnis zu den Risiken der teilnehmenden Patienten. |
| a. Voraussehbarer therapeutischer Nutzen für die Studienteilnehmer (**individueller Nutzen** für den einzelnen Patienten) | Es ist an anderen Patientenpopulationen gezeigt, dass die Patienten unter verbesserter Hämodynamik eine geringere Rate an Komplikationen erleiden und früher in klinisch stabilem Zustand sein können, um nach Hause entlassen werden zu können. Dieser an anderen Populationen nachgewiesene Effekt ist auch der voraussehbare therapeutische Nutzen der Patienten der Protokoll-Gruppen. |
| b. Voraussehbarer medizinischer Nutzen für zukünftig erkrankte Personen **(Gruppennutzen)** | Ziel dieser Studie muss es sein, den Einfluss des erweiterten hämodynamischen Monitorings und die Verwendung von klinischen Behandlungsprotokollen aufgrund von klinischen Studien zu untersuchen, um die Komplikationsrate zu senken.  Da diese Operation sehr häufig (ca. 250 Eingriffe/Jahr in der Klinik für Allgemein-, Visceral- und Transplantationschirurgie, Charité – Universitätsmedizin Berlin, Campus Virchow-Klinikum) und mit einem hohen Anteil an perioperativen Komplikationen verbunden ist, ist der Nutzen von neuen Erkenntnissen zur komplikationsärmeren Durchführung des Eingriffes von hohem Wert für zukünftige Patienten. |
| c. **Risiken** und Belastungen für die Studienteilnehmer (alle im Einzelnen auflisten) | Die Risiken des ösophagealen Dopplers sind eine Verletzungen des Ösophagus und Blutung im Nasen-Rachen-Raum. Die Gefahr einer Ösophagusverletzung ist als gering einzustufen, gibt es laut Deltex-Medical nur einen fraglichen Fall einer Ösophagusverletzung bei über 800.000 Anwendungen der Ösophagussonden. Die Gefahr der Blutungen im Nasen-Rachen-Raum ist höher bei nasaler Applikation der Sonden. Da aber in dieser Studie nur die orale Anwendung durchgeführt wird, ist auch dieses Risiko als gering zu bewerten. Auch haben die bisherigen Studien von keiner relevanten Morbidität durch die ösophageale Sonde berichtet.  Die Belastung der Patienten ist ebenfalls als sehr gering einzustufen. Eine Magensonde ist mit der Ösophagussonde in Größe und Form der Platzierung vergleichbar. Die Anlage einer Magensonde wird unter lokaler Anästhesie gut durch wache Patienten toleriert. Die ösophageale Sonde wird in dieser Studie aber in Narkose platziert und vor Extubation wieder entfernt. Deshalb ist die Belastung als minimal in der Doppler-Gruppe einzustufen.  Für die Patienten der LiDCOrapid-Gruppe entstehen keine zusätzlichen Risiken, denn die Patienten erhalten aufgrund der Operation eine arterielle Blutdruckmessung, dessen Signal lediglich durch den Monitor zusätzlichen Analysen unterzogen wird, um die hämodynamischen Parameter zu bestimmen.  Die vorgesehenen Blutentnahmen werden in gleicher Weise, wie auch die während Ihres Aufenthaltes in der Klinik erforderlichen Blutentnahmen, durchgeführt. In der Regel treten durch die Blutentnahme keine merklichen Einschränkungen des körperlichen Wohlbefindens auf. Vereinzelt können harmlose Kreislaufstörungen auftreten, die durch kurzzeitiges Ruhen nach der Entnahme weitgehend vermeidbar sind. An der Stelle, an der Blut entnommen wird, kann sich ein blauer Fleck bilden. Noch seltener sind Schädigungen von Blutgefässen (Venen, Arterien) und Nerven sowie Entzündungen durch Punktion der Einstichstelle. Eine Übertragung von Infektionskrankheiten ist ausgeschlossen, da ohne Ausnahme sterile, geschlossene Einmalsysteme und Einmalkanülen zur Blutentnahme angewandt werden. Zusätzlich wird die Abnahme der Blutproben aus liegenden Zugängen im Rahmen der klinischen Routine durch geschultes Personal angestrebt. Die Menge an abzunehmendem Blut wird sich bei 20-30 ml pro Tag belaufen über fünf perioperative Tage hinweg. |
| 12. Maßnahmen zur Risikobeherrschung | Die Gefahren für die Platzierung der ösophagealen Sonde bestehen in Ösophagusverletzungen und Blutungen im Nasen-Rachen-Raum. Eventuell vorliegende Pathologien oder kürzlich zurückliegende Eingriffe im Bereich des Ösophagus und des Nasen-Rachen-Raums als Risikofaktoren für eine Komplikation stellen Kontraindikationen für die Studienteilnahme dar.  Die Anwendung der ösophagealen Sonden findet unter strenger Überwachung eines Anästhesisten auf Facharztniveau statt und die Patienten werden bis zum Studienende überwacht. |
| 13. Abbruchkriterien | Unter den nachfolgenden Bedingungen tritt ein vorzeitiges Ausscheiden eines Patienten aus der Studie gemäß der Abbruchkriterien ein:  Die folgenden Ereignisse können als Begründung angesehen werden:   - persönlicher Wunsch des Patienten - jede andere Situation, bei der nach Ansicht des Studienarztes eine weitere Teilnahme an der klinischen Studie nicht im besten Interesse des Patienten sein würde - Entblindung im Falle eines medizinischen Notfalles - signifikante Protokollverletzungen - Nachträgliches Auftreten eines Ausschlusskriteriums   Ein vorzeitiges Ende der Studie bzw. ein Abbruch der gesamten Studie kann aufgrund folgender Umstände in Frage kommen:   - Entscheidung des Studienleiters bei unvertretbaren Risiken unter Nutzen-Risiko-Abwägung - neue (wissenschaftliche) Erkenntnisse während der Laufzeit der klinischen Studie, die die Sicherheit der Studienteilnehmer gefährden können (positive Nutzen-Risiko-Abwägung nicht mehr gegeben) |
| 14. Anzahl, Alter und Geschlecht der betroffenen Personen | Da weder für die primäre als auch für die sekundären Zielkriterien verlässliche Literaturangaben vorliegen, die bei vergleichbaren Eingriffen die Zielkriterien darlegen, ist eine statistische Planung der Fallzahl derzeit unmöglich.  Deshalb handelt es sich bei dieser Studie um eine Pilot-Studie, in die je Gruppe 20 Personen, also insgesamt 60 Patienten eingeschlossen werden. Im Rahmen der vorliegenden Studie erfolgt keine geschlechts-bezogene Auswahl der Studienteil-nehmer.  Es werden Männer und Frauen > 18 Jahre eingeschlossen. |
| 15. Statistische Planung und Angabe sowie biometrische Begründung der Fallzahl und Unterschrift des/der Statistikers/Statistikerin | Für alle Zielgrößen werden die Ergebnisse zunächst exploratorisch untersucht und deskriptiv ausgewertet. Für die primäre Zielgröße kommt wegen der geringen Stichprobenumfänge als konfirmatorische Auswertungsmethode der (exakte) nichtparametrische Kruskal-Wallis-Test, gefolgt von paarweisen (exakten) Mann-Whitney-Tests auf Unterschiedlichkeit zwischen den betrachteten Gruppen in Frage. Wegen des geringen Stichprobenumfangs und möglicher Abweichungen von der Normalverteilung werden für die Überprüfung der sekundären Zielgrößen ebenfalls der nichtparametrische Mann-Whitney U-Test oder der exakte Fisher-Test eingesetzt.  Sämtliche Tests werden mit einem Fehler 1. Art α = 5% (zweiseitig) durchgeführt. Die erzielten p-Werte verstehen sich im Sinne der Pilotstudie als explorativ, d.h. lassen keine konfirmative Verallgemeinerung zu. Aus dem gleichen Grunde erfolgt keine α-Adjustie-rung für multiples Testen.  Da für diese Pilotstudie keine statistische Fallzahl-planung vorgenommen werden kann, ergibt sich die angegebene Patientenzahl (20 pro Gruppe) aus sachlichen Gesichtspunkten und den in der Klinik bestehenden Möglichkeiten zur Durchführung der Pilotstudie. Mit den Ergebnissen der Studie soll im Anschluss eine prospektive randomisierte Studie mit statistischer Fallzahlplanung angeschlossen werden.  Die Auswertung der Studienergebnisse erfolgt sowohl nach dem „intent-to-treat“- als auch nach dem „per-protocol“-Prinzip.  Die numerischen Berechnungen werden mit Hilfe des Statistik Programm-Paketes SPSS, Version 17, Copyright© SPSS, Inc., Chicago, Illinois 60606, USA, durchgeführt. |
| 16.  a. Darlegung und ggf. Erläuterung der **Ein- und Ausschlusskriterien** | **Einschlusskriterien:**   - Durchgeführte Patientenaufklärung und schriftliche Einwilligung - Patienten zur elektiven Leberteilresektion an der Charité, Campus Virchow-Klinikum   **Ausschlusskriterien:**   - Alter < 18 Jahre - Fehlende schriftliche Einwilligungserklärung - Mangelndes deutsches Sprachverständnis - Fehlende Bereitschaft zur Speicherung und Weitergabe von pseudonymisierten Krankheitsdaten im Rahmen der klinischen Studie - Schwangerschaft oder Stillzeit - Teilnahme an einer anderen klinischen Studie während der Teilnahme - Unterbringung in einer Anstalt auf gerichtliche oder behördliche Anordnung - Mitarbeiter der Charité - Unklare Alkohol-Anamnese - Schwere Erkrankung des Ösophagus oder der oberen Luftwege - Operation des Ösophagus oder der oberen Luftwege in den letzten zwei Monaten - Nachgewiesene Koagulopathien (z.B. von Willebrand) - Neurologische / psychiatrische Erkrankung - Bei Krankenhaus-Aufnahme chronische Herzinsuffizienz Stadium NYHA IV - Status nach der American Society of Anesthesiology größer als IV - Renale Insuffizienz mit Abhängigkeit von Hämodialyse - Vorliegen eines pulmonalen Ödems in der Röntgen-Thorax-Aufnahme präoperativ - Intrakranielle Blutung innerhalb eines Jahres vor Aufnahme in die Studie - Allergie gegen Gelatine-Präparate |
| 16.  b. **Teilnehmerinformation** (wer diese mündlich erteilt und Angabe, wie viel Zeit zwischen Aufklärung und Einwilligung verbleibt, ansonsten Verweis auf deren Inhalt als Anlage möglich) | Beilage zum Antrag |
| 16.  c. **Einwilligungserklärung** (Verweis auf deren Inhalt als Anlage möglich) | Beilage zum Antrag |
| 16.  d. Ggf. **Information und Einwilligung des gesetzlichen Vertreters** (ggf. auch Beschreibung des Verfahrens zur Einrichtung einer gerichtlichen Betreuung) | Entfällt |
| 17. Maßnahmen zur Gewinnung von Studienteilnehmern (Aushang ?, Zeitungsannoncen? Etc.) | Es wird das abdominal-chirurgische Krankengut der Charité – Campus Virchow-Klinikum auf Patienten gescreent, die den Einschlusskriterien entsprechen könnten. |
| 18. Ggf.: **Grund für die Einbeziehung und Darlegung des therapeutischen Nutzens für Personen, die minderjährig und/oder nicht einwilligungsfähig sind.** | Entfällt. |
| 19. Beziehung zwischen Studienteilnehmer und Studienarzt/-ärztin (Ist der Studienarzt zugleich der behandelnde Arzt?) | Die Patienten werden eingebunden in die tägliche Routine von den Ärzten der Klinik für Allgemein-, Visceral- und Transplantationschirurgie und Anästhesiologie mit Schwerpunkt operative Intensivmedizin der Charité – Campus Virchow-Klinikum betreut. Aus diesen Kliniken rekrutieren sich auch die Studienärzte dieser Studien. Beide Kliniken verfügen über weitreichende Erfahrung mit klinischen Studien.  Die Patienten können frei über die Teilnahme an der Studie entscheiden, ohne dass ihre Entscheidung zur oder gegen die Teilnahme Einfluss auf ihre klinische Versorgung hätte.  Der Studienarzt ist nicht zugleich der behandelnde Arzt. |
| 20. Ggf. Erklärung zur Einbeziehung möglicherweise vom Sponsor oder Studienarzt abhängiger Personen | Entfällt. |
| 21. Maßnahmen, die eine Feststellung zulassen, ob ein Studienteilnehmer an mehreren Studien zugleich oder vor Ablauf einer in der vorangegangenen Studie festgelegten Frist teilnimmt. | Im Rahmen der Einschlussuntersuchung wird der Patient zu paralleler Teilnahme an einer anderen Studie befragt. Wenn er Teilnehmer einer anderen Studie ist oder eine Studienteilnahme innerhalb der voraussichtlichen Dauer der Teilnahme an dieser Studie geplant ist, liegt ein Ausschlusskriterium für die Teilnahme an der Studie vor. |
| 22. Ggf.: Honorierung bzw. Kostenerstattung der Studienteilnehmer (Höhe, wofür soll gezahlt werden ?) | Entfällt. |
| 23. Ggf.: Plan für die Weiterbehandlung und medizinische Betreuung der betroffenen Personen nach dem Ende der Studie | Die Studie dauert vom Operationsbeginn bis zum ersten postoperativen Tag. Nach der Operation erfolgt eine Überwachung der Patienten über den klinischen Aufenthalt hinweg bis zur Entlassung (postoperativ < 8 Tage) oder bis zum 8. postoperativen Tag. Da der Patient nur nach Erfüllung definierter Entlassungskriterien aus dem Krankenhaus entlassen wird, ist damit die Nachsorge nach der perioperativen Studie gewährleistet.  Die Daten dieser Studie werden entblindet, nachdem beim letzten Patienten die Untersuchung erfolgt ist. |
| 24. Ggf.: Versicherung der Studienteilnehmer (Versicherungsbestätigung und Versicherungsbedingungen, Versicherer, Versicherungsumfang, Versicherungsdauer) | Der Ethikantrag, die Patienteninformation und die Einwilligungserklärung sind per E-Mail an Frau E. Eckert zur Bestätigung der Betriebshaftpflicht des Krankenhauses für die Studie gesandt worden. |
| 25. Ggf.: Dokumentationsverfahren (Verweis auf CRF-Bögen möglich) | Die Datenerhebung wird mittels papierbasiertem Case Report Form durchgeführt.  Die Patientendaten werden nur pseudonymisiert erfasst und abgespeichert. Der Studienarzt generiert für jeden neu anzulegenden Patienten gemäß den unten aufgeführten Richtlinien ein Pseudonym erstellen. Die eindeutige Zuordnung zum Patienten erfolgt über die Patienten-Identifikationsliste, welche sicher aufbewahrt werden muss.  Das Pseudonym wird aus den ersten zwei Anfangs-buchstaben der Studie, der Zentrumsnr. CVK=1, CCM=2, und einer laufenden, dreistelligen Nummer gebildet.  Beispiel für den ersten Patienten der Studie:  LE 1 001 |
| 26. Ggf.: Beschreibung, wie der Gesundheitszustand gesunder betroffener Personen dokumentiert werden soll | Der Einschluss von Patienten erfolgt aus einer Patientenpopulation mit einer zugrunde liegenden Erkrankung, aufgrund derer eine Leberteilresektion indiziert ist. Es werden keine gesunden Patienten in diese Studie eingeschlossen. |
| 27. Ggf.: Methoden, unerwünschte Ereignisse festzustellen, zu dokumentieren und mitzuteilen (wann, von wem und wie ??) | Die Patienten werden intensiv während der Operation durch die Anästhesie überwacht und verlassen den operativen Bereich erst, wenn die Entlassungskriterien im Aufwachraum oder die Entlassungskriterien der Intensivpflegestation erfüllt sind. Neben der routinemäßigen täglichen Betreuung durch die Ärzte der Klinik für Allgemein-, Visceral- und Transplantationschirurgie erfolgt eine Visite durch die Studienärzte der Universitätsklinik für Anästhesiologie mit Schwerpunkt operative Intensivmedizin, um nach unerwünschten Ereignissen zu schauen. Die Dokumentation von schwerwiegenden unerwünschten Ereignissen erfolgt zur Meldung beim Studienleiter innerhalb von 24 Stunden. Da eine Leberteilresektion einen Eingriff mit einem erhöhtem Risiko darstellt, ist die Abweichung von Parametern von der Norm als häufig anzusehen. Aufgrund dessen wird gemäß klinischen Gesichtspunkten eine Liste von Ausnahmen für die meldepflichtigen unerwünschten Ereignisse erstellt, da sie regelmäßig in der klinischen Routine im Zusammenhang mit Leberteilresektionen auftreten und nicht als hinweisend auf ein unerwünschtes Ereignis durch die Studien-Protokolle anzusehen sind. Für die perioperativen Blutabnahmen wird eine Plausibilitätsliste erstellt, die die Meldung von pathologischen Werten und die Grenzwerte als meldepflichtig regelt. Die postoperative Überwachung im Rahmen der Krankenhausversorgung wird fortgeführt bis zum Tag der Entlassung (postoperativ < 8 Tage) oder bis zum 8. postoperativen Tag. |
| 28. Vorgehen zum Schutz der Geheimhaltung der gespeicherten Daten, Dokumente und ggf. Proben, Darlegung der Verschlüsselung der Daten von Studienteilnehmern (*bitte nicht Initialen und Geburtsdatum als Codierungsschema verwenden!)* | Für jeden Patienten wird ein Pseudonym gebildet und in der Patientenidentifikationsliste den persönlichen Daten des Patienten zugeordnet. Diese wird verschlossen aufbewahrt. Die Patientendokumentation erfolgt nur mit dem Pseudonym.  Das Pseudonym wird aus den ersten zwei Anfangs-buchstaben der Studie, der Zentrumsnr. CVK=1, CCM=2, und einer laufenden, dreistelligen Nummer gebildet.  Beispiel für den ersten Patienten der Studie:  LE 1 001 |
| 29. Erklärung zur Einhaltung des Datenschutzes | Der Studienarzt wird die erhobenen Daten und die personenbezogenen Daten für Zwecke der Verwaltung und Durchführung der Studie sowie für Zwecke der Forschung und statistischen Auswertung verwenden.  Für jegliche Weitergabe von Daten an Beteiligte an der Studie werden die Daten mit einer Codenummer versehen (Pseudonymisierung der Daten). Auf den Codeschlüssel, der es erlaubt, die studienbezogenen Daten mit dem Patienten in Verbindung zu bringen, haben nur der Studienarzt und seine Mitarbeiter Zugriff. Sämtliche Aufzeichnungen, anhand derer die Patienten identifiziert werden können, werden streng vertraulich behandelt. |
| 30. Namen und Anschriften der Einrichtungen, die als Studienzentrum oder Studienlabor in die Studie eingebunden sind, sowie der Studienleiter und die Studienärzte | Studienleitung:  Prof. Dr. Claudia Spies  Weitere Studienärzte:  Dr. Aarne Feldheiser  Ansgar Jones  Dr. Nikola Magheli  André Haas  Dr. Vera von Dossow  Dr. Christian Pille  Universitätsklinik für Anästhesiologie und operative Intensivmedizin,  Charité – Universitätsmedizin Berlin,  Charité Campus Mitte  Charitéplatz 1, 10117 Berlin  Charité Campus Virchow Klinikum  Augustenburger Platz 1, 13353 Berlin  Tel.: +49 (0) 30 450 551 001 / 12  Fax: +49 (0) 30 450 551 900  E-Mail: [anaesthesie-virchow-klinikum@charite.de](mailto:anaesthesie-virchow-klinikum@charite.de)  Prof. Dr. Peter Neuhaus,  PD Dr. Ulf Neumann  Dr. M. Stockmann  Dr. M. Bahra  Klinik für Allgemein-, Visceral- und Transplantationschirurgie  Charité – Universitätsmedizin Berlin  Charité Campus Virchow Klinikum  Augustenburger Platz 1, 13353 Berlin  Tel.: +49 (0) 30 450 55 20 01  Fax: +49 (0) 30 450 55 29 00  E-Mail: ulf.neumann@charite.de  Dr. med. Andreas Weimann  Zentralinstitut für Laboratoriumsmedizin und Pathobiochemie  Campus Virchow-Klinikum  Augustenburger Platz 1, 13353 Berlin  Tel.: +49-30-450 569032  Fax: +49-30-450 569900  E-Mail: andreas.weimann@charite.de |
| 31. Angaben zur Eignung der Prüfstelle, insbesondere zur Angemessenheit der dort vorhandenen Mittel und Einrichtungen sowie des zur Durchführung der klinischen Prüfung zur Verfügung stehenden Personals und zu Erfahrungen in der Durchführung ähnlicher Studien | Die Charité – Campus Virchow-Klinikum ist ein Krankenhaus der Maximalversorgung und besitzt eine 24-stündige Bereitschaft zur Patientennotfallversorgung. Die Kliniken für Allgemein-, Visceral- und Transplantationschirurgie und Anästhesiologie mit Schwerpunkt operative Intensivmedizin der Charité – Campus Virchow-Klinikum besitzen weitreichende Erfahrungen mit der Durchführung von klinischen Studien. |
| 32. Vereinbarung über den Zugang des Prüfers/Hauptprüfers/Leiter der klinischen Prüfung, zu den Daten und den Grundsätzen über die Publikation | Die Aufzeichnungen der Patientendaten werden streng vertraulich behandelt und die Weitergabe der Daten wird nur in pseudonymisierter Form erfolgen. Der Studienleiter hat Zugang zu sämtlichen Daten. Die Studienärzte und weiteres Studienpersonal haben nur insofern Zugang zu den personenbezogenen Daten, wie es zur Durchführung der Studie notwendig ist.  Die Publikation wird unabhängig vom Ergebnis der Studie in einem Peer-Reviewed Journal angestrebt. |
| 33. **Angaben zur Finanzierung der Studie**  (wir weisen auf § 263 StGB hin)  a. Finanzierungsquelle (Name und Sitz) | Charité – Universitätsmedizin Berlin  Finanzierung im Rahmen der inneruniversitären Forschungsförderung |
| 33.  b. Höhe der kalkulierten Kosten pro Teilnehmer und insgesamt | Kosten einer ösopheagealen Doppler-Sonde: etwa 120,00 €, die Analysekarten zur Nutzung des LiDCOrapid werden von der Firma gestellt.  Die zerebralen Oximeter werden von Somanetics Corporation und die InSpectra StO2-Sensoren zur Messung der Gewebeoxigenierung werden von Hutchinson Technology gestellt. Die Laborkosten belaufen sich auf etwa 60,00 € pro Teilnehmer. |
| 33.  c. Höhe der Kostenerstattung pro Teilnehmer und insgesamt | Keine Kostenerstattung der Teilnehmer. |

Name und Unterschrift der Antragstellerin:

Ich versichere hiermit, dass die in diesem Antrag gegebenen Informationen richtig sind. Ich bin der Auffassung, dass es möglich ist, die o.g. Studie in Übereinstimmung mit dem Protokoll, den nationalen Rechtsvorschriften durchzuführen.

Mir ist bekannt, dass ich gemäß §19 Berliner Datenschutzgesetz (BlnDSG) verpflichtet bin, für automatisierte Verarbeitungen personenbezogener und personenbeziehbarer Daten eine Datei- und Verfahrensbeschreibung zu erstellen und diese gemäß §19a dem behördlichen Datenschutzbeauftragten der Charité zur Verfügung stellen muss. Ich bin darüber informiert, dass wenn es sich um ein Verfahren handelt, mit dem Daten verarbeitet werden, die einem Berufsgeheimnis (z.B. ärztliche Schweigepflicht) unterliegen, ich gemäß §5 BlnDSG vor dem Einsatz dieses Verfahrens eine Vorabkontrolle durch den behördlichen Datenschutzbeauftragten der Charité veranlassen muss und ich das Verfahren erst bei positivem Prüfergebnis anwenden darf.

Name: Prof. Dr. med. Spies

Vorname: Claudia

Adresse: Augustenburger Platz 1

Position: Direktorin der Klinik für Anästhesiologie und operative Intensivmedizin, Charité – Campus Mitte und Campus Virchow-Klinikum

Ort, Datum: Berlin, den

Unterschrift:

**Anhang 1:**

**Auflistung einer Auswahl an outcome-relevanten Studien mit dem ösophagealen Doppler:**

| **Studie** | **n-Zahl**  **ASA-Status** | **Chirurgischer Bereich** | **Outcome der Patienten** |
| --- | --- | --- | --- |
| Mythen  1995 ^6^ | 60  ASA III | Kardioanästhesie | Keine größere Morbidität  41% Reduktion ITS-Behandlung  37% Reduktion Krankenhaus-Behandlung |
| Sinclair  1997 ^7^ | 40  ASA II/III | Hüft-Frakturen Traumatologie | 39% Reduktion Krankenhaus-Behandlung |
| Gan  1999 ^8^ | 44 | Urologie/  Gynäkologie | 33% Reduktion Krankenhaus-Behandlung |
| Venn  2002 ^9^ | 90  ASA III/IV | Hüft-Frakturen Traumatologie | 44% Reduktion der Zeit bis zur medizinischen Fitness  23% Reduktion Krankenhaus-Behandlung |
| McKendry  2000 ^10^ | 39 | Kardioanästhesie | 19% Reduktion Krankenhaus-Behandlung |
| Gan  2002 ^8^ | 100  ASA I/II/III | Urologie/  Gynäkologie | 29% Reduktion Krankenhaus-Behandlung |
| Wakeling  2005 ^11^ | 134  ASA I-III | Abdominal-Chirurgie | 37 % Reduktion der Patienten mit Komplikation  13 % Reduktion Krankenhaus-Behandlung |
| Noblett  2006 ^12^ | 108 | Abdominal-Chirurgie | 22 % Reduktion Krankenhaus-Behandlung  Reduktion der lebensgefährlichen Komplikationen |

**Abkürzungen der Tabelle:**

ASA American Society of Anesthesiologists

ITS Intensivpflegestation

**Anhang 2:**

|  | | | **Differenzen zu Kontroll-Technik** | | | **Limits of Agreement** | |
| --- | --- | --- | --- | --- | --- | --- | --- |
| **Studien und die Kontroll-Techniken** | **No. Pat.** | **No.**  **Obs.** | **Bias**  **L/min** | **Präzision**  **L/min** | **95% Ko.-In.**  **L/min & %** | **Oberes**  **L/min** | **Unteres**  **L/min** |
| de Wilde et al. ^13^  Kardiochirurgie, intra-op.  CO-Thermodilution | 27  88 | 199  301 | -0.17  -0.02 | 0.69  0.65 | 1.38 (28.6%)  1.3 (25.7%) | -1.55  -1.28 | 1.20  1.32 |
| Missant et al. ^14^  Kardiochirurgie, intra-op.  CO-Thermodilution | 20 | 149 | -0.03 | 0.65 | 1.3  (29%) | -1.33 | 1.26 |
| Costa et al. ^15^  Lebertransplantation, post-op.  CO-Thermodilution | 23 | 151 | 0.29 | 1.09 | 2.17  (16.8%) | -1.87 | 2.46 |
| Kim et al. ^16^  Kinder-ITS (*: Herzindex)  CO-Thermodilution | 20 | 73 | 0.19* | 0.14* | 0.28*  (8.5%) | -0.09* | 0.47* |
| Pittman et al. ^17^  Intensivstation (24 hrs.)  Lithium-Thermodilution | 21 | 83 | -0.01 | 0.82 | 1.64  (27%) | -1.65 | 1.63 |
| Hamilton et al. ^18^  Kardiochirurgie, post-op.  Lithium-Thermodilution | 20 | 80 | 0.1 | 0.6 | 1.2  (21.8%) | -1.1 | 1.3 |

**Abkürzungen der Tabelle:**

No. Anzahl

Pat. Patienten

Obs. Untersuchungen

Ko.-In. Konfidenz-Intervall

**Anhang 3:**

**Algorithmus für die zielorientierte Volumen- und Katecholamintherapie der Interventionsgruppen:**

*: Das Schlagvolumen nach der initialen Vorlast-Optimierung ist der Ausgangs-Schlagvolumenindex.

^#^: Der Abfall des Schlagvolumenindex ist in Bezug zum Ausgangs-Schlagvolumenindex zu sehen.

^x^: Findet sich klinisch (durch die Aussage des Chirurgen in Abstimmung mit dem Anästhesisten) oder hämodynamisch messbar (in der Form eines erhöhten zentral-venösen Druckes in Verbindung mit dem klinischen Eindruck) ein Rückstau in die Leber, soll die Gabe von Enoximon (Initial-Dosis: 2,5µg/kg/min, steigerbar bis 10µg/kg/min bzw. eine Bolusapplikation von 1µg/kg) bzw. die Gabe von Nitro-Präparaten entsprechend der klinischen Abwägung des behandelnden Anästhesisten erfolgen. Die gleichzeitige Gabe von beiden Präparaten darf am selben Patienten wegen der Gefahr einer Hypotonie nicht erfolgen. .

**Abkürzungen des Diagramms:**

SVI Schlagvolumenindex

SBP Systolischer Blutdruck

MAP Mittlerer Arterieller Blutdruck

**Anhang 4:**

**Definition der infektiösen Komplikation:**

Für die Diagnose einer Infektion gelten folgende SOP aus unserem Hause gemäß den CDC-Kriterien und der ATS-Guidelines ^19^:

**Pneumonie:**

Dabei muss eines der folgenden Kriterien erfüllt sein:

**Rasselgeräusche bei Auskultation oder Dämpfung bei Perkussion**

und eines der folgenden Anzeichen:

- Neues Auftreten von eitrigem Sputum oder Veränderung der Charakteristika
- Mikroorganismen aus der Blutkultur isoliert
- Krankheitserreger aus Bronchoalveolärer Lavage, Bronchialabstrich, transtrachealem Aspirat oder Biopsieprobe isoliert

**Röntgenuntersuchung des Thorax zeigte neues oder progessives Infiltrat, Verdichtung, Kavitation oder pleuralen Erguss**

und eines der folgenden Zeichen:

- Neues Auftreten von eitrigem Sputum oder Veränderung der Charakteristika
- Mikroorganismen aus Blutkultur isoliert
- Krankheitserreger aus Bronchoalveolärer Lavage, Bronchialabstrich, transtrachealem Aspirat oder Biopsieprobe isoliert

**Tracheobronchitis:**

Ohne Anzeichen der Pneumonie müssen **zwei** der anschließenden Kriterien:

- Fieber (38°C)
- Husten
- Neue oder erhöhte Sputumproduktion
- Trockene Rasselgeräusche
- Giemen

und **eines** der folgenden Kriterien erfüllt sein:

- Kulturelle Isolierung eines Mikroorganismus aus dem Trachealsekret oder dem bei der Bronchoalveolären Lavag gewonnenen Material
- Positiver Antigen-Test in den Atemwegen

**Wundinfektion:**

**Oberflächliche postoperative Wundinfektion:**

Infektionen an der Inzisionsstelle innerhalb von 30 Tagen nach der Operation, die nur Haut oder sukutanes Gewebe mit einbezieht,

und es ist **eines** der folgenden Kriterien erfüllt:

- Eitrige Sekretion aus der oberflächlichen Inzision
- Kultureller Nachweis eines Mikroorganismus aus einem aseptisch entnommenen Wundsekret oder Gewebekultur von der oberflächlichen Inzision

Eines der folgenden Anzeichen:

- Schmerz
- Empfindlichkeit
- lokalisierte Schwellung
- Rötung oder Überwärmung
- Der Chirurg öffnet die oberflächliche Inzision bewusst, es sei denn, es liegt eine negative Kultur vor Diagnose des Chirurgen.

**Tiefe postoperative Wundinfektion:**

Infektion innerhalb von 30 Tagen nach der Operation. Die Infektion scheint mit der Operation in Verbindung zu stehen und erfasst Faszienschichten und Muskelgewebe.

Zudem muss **eines** der folgenden Kriterien erfüllt sein:

- Eitrige Sekretion aus dem tiefen Einschnitt, aber nicht aus dem Organ bzw. Raum
- Spontan oder vom Chirurgen bewusst geöffnet, wenn der Patient mindestens eines der nachfolgenden Symptome hat:
  - Fieber (>38°C)
  - lokalisierter Schmerz oder Empfindlichkeit
  - es liegt eine negative Kultur vor
- Abszess oder sonstige Zeichen einer Infektion sind bei der klinischen Untersuchung, während der erneuten Operation, bei der histopathologischen Untersuchung oder bei radiologischer Untersuchung ersichtlich
- Diagnose des Chirurgen

**Harnwegsinfektion:**

Für die Diagnose einer Hanwegsinfektion muss **eines** der folgenden erfüllt sein:

- Fieber (>38°C)
- Harndrang
- Häufigkeit
- Dysurie oder suprapubische Missempfindungen

und eine Urinkultur von >10^5^ Kolonien/ml Urin mit nicht mehr als zwei Arten von Mikroorganismen.

Oder **zwei** der anschliessenden:

- Fieber (>38°C)
- Harndrang
- Häufigkeit
- Dysurie oder suprapubische Missempfindungen

und **eines** der nachfolgend aufgelisteten Anzeichen:

- Harnteststreifen für Leukozytenesterase und/oder Nitrat positiv
- Pyurie (>10 weiße Blutkörperchen/ml^3^ oder >3 Leukozyten/Gesichtsfeld bei starker Vergrößerung im nicht-zentrifugiertem Urin)
- Bei Gram-Färbung einer nicht-zentrifugierten Urinprobe Nachweis von Mikroorganismen.
- zwei Urinkulturen mit wiederholter Isolierung des gleichen Uropathogens mit >10^2^ Kolonien/ml Urin im Katheterurin
- Urinkultur mit >10^5^ Kolonien/ml Urin einzelner Uropathogene bei Patienten, die mit der entsprechenden antimikrobiellen Therapie behandelt werden

**Sinusitis:**

Für diese Diagnosestellung muss gemäß den CDC-Kriterien **eines** der folgenden Kriterien erfüllt sein:

- **Entweder** Isolation eines Mikroorganismus aus der Kultur von eitrigem Material, das aus der Nebenhöhle entnommen wurde
- **oder** eines der folgenden Anzeichen:
  - Fieber (>38°C)
  - Schmerz oder Empfindlichkeit im Bereich der betroffenen Nebenhöhle
  - Kopfschmerz, eitriges Exsudat oder Obstruktion der Nase
- **und:**
  - Diaphanoskopie positiv oder
  - Röntgenologischer Infektionsnachweis

**SIRS:**

Sepsis wird entsprechend den Kriterien der „Society of Critical Care Medicine Consensus Conference“ definiert:

Mindestens **zwei** Kriterien für die Definition von SIRS vorhanden sein:

- Körpertemperatur > 38,0 °C oder < 36,0 °C
- Herzfrequenz: >90/min ohne ß-Blockade
- Oder **Eines** der folgenden Zeichen für respiratorische Insuffizienz:
  - Atemfrequenz: <20/min
  - Hyperventilation P_a_CO_2_ <32mmHg bei Spontanatmung
  - p_a_O_2_ < 70 mmHg (bei Spontanatmung)
  - p_a_O_2_/F_i_O_2_ < 175 (bei maschineller Beatmung und fehlender pulmonaler Vorerkrankung)
- Körpertemperatur: >38°C oder <36°C
- Leukozytenzahl: >12000/mm^3^ oder <4000/mm^3^ oder >10% unreife Neutrophile

**Sepsis:**

- SIRS-Kriterien (s.o.) sind erfüllt
- Eine vermutete oder nachgewiesene Infektionsquelle
- bzw.
- eine überschießende Aktivierung von primär protektiven Defensivsystemen des septischen Patienten im Sinne einer host defence failure disease

**Schwere Sepsis:**

Sepsis-Kriterien und Systemische Toxizität oder schlechte Organperfusion **mit zwei oder mehr** charakteristischen Merkmalen:

- - Akutes Nierenversagen (Oligurie < 0,5 ml*kg^-1^*h^-1^)
  - Erhöhtes Plasmalaktat (>1,8 mmol/l)
  - CI >4,0 l/min mit SVR <800 dyn*s*cm^-5^
  - Metabolische Azidose (pH <7,3 oder Basendefizit >5)
  - Arterielle Hypoxie: P_a_O_2_ <75 mmHg (<10kPa)
  - Thrombozytenzahlabfall innerhalb der letzten 24h ohne anderweitige Erklärung (<100000/ml oder Abfall von 50% vom Ausgangswert)
  - Abnorme Gerinnungswerte innerhalb der letzten 24h ohne anderweitige Erklärung (Quick >1,5 oder PTT >1,2*Kontrollwert)
  - Plötzlicher Abfall des Glascow-Coma-Scale

**Septischer Schock:**

Zeichen von Sepsis und arterieller Hypotension trotz ausreichender Volumensubstitution, begleitet von verminderter Organperfusion oder Organdysfunktion:

Hypotension, die anhand eines der folgenden Kriterien definiert wurden:

- - Systolischer Blutdruck <90 mmHg
  - Anhaltender Blutdruckabfall von 40 mmHg mit entsprechendem Flüssigkeitsbedarf ohne Antihypertensiva
  - Therapie mit Vasopressoren, um den Blutdruck über 90 mmHg zu halten

**ARDS Definition nach amerikanisch-europäischer Consensus-Konferenz (AECC):**

- Akutes Auftreten der Erkrankung
- paO2/FiO2 (Horovitz)-Quotient < 200 mmHg unabhängig vom verwendeten PEEP
- bilaterale Infiltrate im a.p.-Röntgen-Thoraxbild
- PCWP < 18 mmHg 🡺 Ausschluss eines kardialen Lungenödems, ggf. auch mittels transösophagealer Echokardiographie

**Lung Injury Score (LIS) von Murray (1988):**

- Ziel des Scores ist die Beurteilung der Lungenfunktion z.B. beim ARDS
- 4 Kriterien:
  - Röntgenbefunde der Lunge
  - Beurteilung nach der Anzahl der Quadranten mit alveolärer Verschattung
    - 0 – 4 Quadranten
  - Hypoxämie/Oxygenierungsstörung beurteilt nach Horovitz-Index:
    - > 300 mmHg
    - 225 – 299 mmHg
    - 175 – 224 mmHg
    - 100 – 174 mmHg
    - < 100 mmHg
  - PEEP:
    - < 5 cm H_2_O
    - 6 – 8 H_2_O
    - 9 – 11 H_2_O
    - 12 – 14 H_2_O
    - > 15 H_2_O
  - Compliance:
    - > 80 ml/cm H_2_O
    - 60 – 79 ml/cm H_2_O
    - 40 – 59 ml/cm H_2_O
    - 20 – 39 ml/cm H_2_O
    - < 19 ml/cm H_2_O

**Punkte:**

0 – 4

0

1

2

3

4

0

1

2

3

4

0

1

2

3

4

**Beurteilung:**

Nach Summe der Gruppenwerte, dividiert durch die Anzahl der beurteilten Gruppen:

Punkte:

0 keine Lungenschädigung

0,1 – 2,5 leichte bis mäßige Lungenschädigung

>2,5 schwere Lungenschädigung (wie z.B. ARDS)

**Anhang 5**

**Definition der RIFLE-Kriterien zur Beschreibung der akuten Niereninsuffizienz ^20^:**

**Anhang 6**

**Definition des Delirium Detection Scores, des Nursing Delirium Rating Scale und des CAM-ICU zur Erfassung und die Behandlung akuter deliranter Zustände:**

**Delirium Detection Score = DDS**

| **Orientierung** | # 0: orientiert zu Person, Ort, Zeit,  Fähigkeit zur Konzentration  # 1: nicht sicher orientiert zu Ort/Zeit,  Unfähigkeit zur Konzentration  # 4: nicht orientiert zu Ort und oder Zeit  # 7: nicht orientiert zu Ort, Zeit und Person |
| --- | --- |
| **Halluzinationen** | # 0: normale Aktivität  # 1: gelegentlich leichte Halluzinationen  # 4: permanent leichte Halluzinationen  # 7: permanent schwere Halluzinationen |
| **Agitation** | # 0: normale Aktivität  # 1: leicht gesteigerte Aktivität  # 4: moderate Unruhe  # 7: schwere unruhe |
| **Angst** | # 0: keine  # 1: leichte Angst  # 4: gelegentlich moderate Angst  # 7: Panikattacken |
| **Schweißausbrüche** | # 0: keine  # 1: meist unbemerkt, v.a. Hände  # 4: Schweißperlen auf der Stirn  # 7: starkes Schwitzen |

**Nursing Delirium Rating Scale = Nu-DESC**

| **Symptom** | **Intensität: 0-2 Punkte** |
| --- | --- |
| **1 Desorientierung**  Desorientierung zu Zeit und oder Ort  Falsche Einordnung der umgebenden Personen |  |
| **2 Unangemessenes Verhalten**  z.B. Ziehen an Kathetern, unkooperatives Verhalten,  Selbstgefährdung durch Uneinsichtigkeit,  Missverstehen der Situation |  |
| **3 Unangemessene Kommunikation**  Unpassendes Kommunikation zu Ort und Situation,  z.B. zusammenhanglose- oder gar keine Kommunikation,  unverständliche sprachliche Äußerungen |  |
| **4 Illusionen / Halluzinationen**  Sehen und oder Hören nicht vorhandener Dinge,  Verzerrung optischer Eindrücke |  |
| **5 Psychomotorische Retardierung**  Verlangsamte Antworten / Reaktionen,  wenige oder keine spontane Äußerung / Aktivität  auch auf Stimulation  Bewusstseinsstörung (RASS < -1) |  |

Bei einer Gesamtpunktzahl > = 2 hat der Patient ein Delir

**CAM-ICU**

**POSTITIV NEGATIV**

**Akuter Beginn oder fluktuierender Verlauf**:

(positiv bei einer „ja“ Antwort):

**A** hat sich der mentale Status verändert? ja nein

(Baseline zuerst definieren)

**oder**

**B** zeigten sich wechselnde mentale Zustände ja nein

in den letzten 24h im Sinne eines veränderten

RASS, GCS oder DDS?

**Unaufmerksamkeit**

(positiv bei > 2 falschen Antworten von 10):

**A** Vorlesen einer Buchstabenreihe; der Patient soll > 2 < 2

bei „A“ die Hand drücken

>>“Minuspunkt“ bei nicht- oder falsch-drücken:

A N A N A S B A U M

**oder**

**B** Zeigen von 5 Bildern (je 3 s), dann zeigen von >2 < 2

diesen 5 und 5 anderen; der Patient soll die ersten

Bilder wiedererkennen

**Unorganisiertes Denken**

(positiv bei > 2 falschen Antworten von 5): > 2 < 2

A 1. schwimmt ein Stein auf Wasser?

2. Schwimmen Fische im See?

3. Wiegt 1 Kilo mehr als 2 Kilo?

4. Kann man mit einem Hammer einen Nagel einschlagen?

**und**

B 1.Halten Sie x Finger hoch, nun dasselbe mit der anderen Hand

(Zahl nicht wiederholen, gibt für beides zusammen 1 Pkt.)

**Veränderte Bewusstseinslage**

Positiv, wenn RASS nicht = 0

**GESAMTURTEIL:** **ja nein**

**wenn 1+2 und entweder 3 oder 4 positiv ist, hat der Patient ein Delir**

**Copyright 2002, E.Wesley Ely, MD, MPH, und Vanderbilt University, all rights reserved**

**SOP Delirtherapie der Klinik für Anästhesiologie und operative Intensivmedizin, CVK, Charité - Universitätsmedizin Berlin:**

Zwingende Voraussetzung ist Diagnose und Therapie der zugrunde liegenden Ursache (Infektion, Entzug, Hypoxie, Elektrolytentgleisung, metabolische Entgleisung, Toxine, Medikamente, Trauma oder ZNS Affektion).

Parallel dazu erfolgt die Delir-Therapie symptomorientiert:

Agitiertes Delir:

- produktive Psychose > Neuroleptika (Haloperidol, Risperidon)
- Angst, Agitation > Benzodiazepine (Lorazepam)
- Agitation, vegetative Symptome > Clonidin

Hypoaktives Delir: > niedrig dosiert Haloperidol, Risperidon

**Anhang 7:**

**Visuelle Analog Skala (VAS) / Numerical Rating Scale (NRS)**

- Erforderlich bei allen Patienten die auf die Frage nach Schmerzen antworten können
- angestrebter Zielwert ≤ 3

| **VAS/NRS** | **Bewertung** | **Punkte** |
| --- | --- | --- |
| Frage nach Schmerzen | Kein bis  maximal vorstellbarer Schmerz | 0 bis  10 |
