# Supplementary material for: Haemodynamic Optimization by Oesophageal Doppler and Pulse Power Wave Analysis in Liver Surgery: A Randomised Controlled Trial
Source: PLoS One. 2015 Jul 17;10(7):e0132715. doi: 10.1371/journal.pone.0132715 (PMC4505861; doi:10.1371/journal.pone.0132715)
Supplement: S2 Protocol — (DOC) [file pone.0132715.s005.doc]

Application for advice from the Ethics committee regarding the execution of a medical-scientific project which doesn’t involve clinical drug testing.

| 1.  a. Title of the study | A pilot study of a goal-directed haemodynamic protocol comparison by monitoring with LiDCOrapid or oesophageal Doppler and conventional therapy during liver resection |
| --- | --- |
| 1.  b. Short title of the study | Haemodynamics in liver resections |
| 2. Ethics committee application number | Will be assigned by the Ethics committee |
| 3.Decisions of other ethics committees in similar cases | Not applicable |
| 4.Subject of the study and its aims  (Hypothesis divided into primary and secondary hypotheses) | Primary Hypothesis:  Compared to conventional therapy an intra-operative goal-directed haemodynamic management by monitoring with LiDCOrapid or oesophageal Doppler improves haemodynamics measured by stroke volume (SV) in patients undergoing elective liver resection.  Secondary hypothesis 1:  Compared to conventional therapy an intra-operative goal-directed haemodynamic management by monitoring with LiDCOrapid or oesophageal Doppler reduces the incidence of perioperative dysfunction (cerebral, pulmonary, renal, abdominal and cardiovascular), the amount of intraoperative blood loss and the postoperative incidence of infections and quality of life in patients undergoing elective liver resection.  Secondary hypothesis 2:  Compared to conventional therapy an intra-operative goal-directed haemodynamic management by monitoring with LiDCOrapid or oesophageal Doppler the postoperative liver function in patients undergoing elective liver resection.  Study groups:  The administration of the volume and catecholamine therapy is carried out by:  1.) Conventional standard therapy  2.) Goal-directed haemodynamic management by monitoring with oesophageal Doppler  3.) Goal-directed haemodynamic management by monitoring with LiDCOrapid  Primary outcome measure:  Stroke volume before intra-operative start of liver resection  Secondary outcome measures:   - Intra-operative and post-operative haemodynamic parameters - Intra-operative and post-operative blood loss - frequency of organ dysfunctions (cerebral, pulmonal, renal, abdominal, cardiovascular) - Post-operative liver function (LiMAx-test, ICG-Clearance, liver Doppler, laboratory tests: enzymatic and chemical parameters) - Peri-operative weight change - Post-operative incidence of infections - Satisfaction of the patients, the surgeons and anaesthetists - Time to fulfilling discharge criteria - Length of intensive care stay and hospital stay (LOS) - Quality of life measure (EQ-5D) - Laboratory tests: peri-operative endothelial and immunological alterations |
| 5. Purpose of the study | The haemodynamic management of patients undergoing liver resections by anaesthesiology is determined by the goal to minimize the intraoperative bleeding during the surgical incision of the liver . This goal is reached by a hypovolaemic volume and fluid strategy to reach a low central-venous pressure. Thereby the backwards flow of the blood from the right heart by the liver veins to the resection surface of the liver is minimized and the blood loss will be reduced. This management is constituted by studies of the 1990ies as a possibility to reduce perioperative morbidity and mortality .  In recent years the surgical patient population changed due to a higher number of comorbidities especially of cardiovascular diseases. According to our clinical experiences this implies that some patients show a substantial drop of stroke volume and cardiac output with consecutive decreased perfusion especially of the splanchnic area induced by this restrictive fluid and volume therapy.  With the oesophageal Doppler and the LiDCOrapid there are two minimal-invasive monitors to offer valid measurements of stroke volume and therefore offer the possibility to guide volume and catecholamine therapy. In colorectal and gynaecological-urological resections and in traumatology the benefit of advanced monitoring on hospital length of stay is well documented in the literature (table of studies in appendix 1). But these studies direct on an intraoperative optimization of cardiac preload according to the Frank-Starling mechanism. For liver resections this approach is not feasible due to the increased risk of intraoperative bleeding. Therefore we created an algorithm for a restrictive volume management by maintaining cardiac output and splanchnic perfusion without preload optimization during the course of surgery. According to our knowledge up to date there is no algorithm in the literature for a goal-directed, restrictive volume management during liver surgery.  The goal of this study is to compare the developed intraoperative, restrictive, goal-directed volume protocol based on advanced haemodynamic monitoring versus standard of care to evaluate the impact on stroke volume as primary outcome measure and to further evaluate the impact on the influence on surgical proceedings and postoperative parameters as secondary outcome measures.  A further goal of this study is with the collaborations of the colleagues of the clinic of general, visceral and transplantation surgery to describe the particular perioperative modules and by their standardization to increase the quality and transparency of the study results. |
| 6. Which of the following regulations apply -   1. Medicinal product law -in accordance with § 20 MPG (equipment does not possess a certificate of conformity or does possess such a certificate but another indication is examined or it will be more invasive or other demanding investigations will be performed) or   - in accordance with § 23MPG?   1. Radiation protection regulation § 23 2. X-ray regulation § 28 a 3. Genetic engineering law 4. Data protection acts | Data privacy act |
| 7. If necessary: Designation and chacharacteristics of Test Products (e.g. devices with MPG studies, please attach information as an appendix) | Not applicable |
| 8. Main results of prior clinical tests or reasons for not performing such tests | The methods of minimal-invasive monitoring of the circulatory parameters are sufficiently evaluated for their use in human beings. The studies are listed in appendix 1 and 2 and are further mentioned in section 9. Due to these explanations we set aside displaying preclinical studies. |
| 9. Main content and results of previous studies/applications of the products to be examined in this study | The oesophageal Doppler is tested in clinical studies since approximately 15 years. A selection of studies is outlined in appendix 1. The oesophageal Doppler in combination with a volume protocol based on his values could show a benefit in cardiovascular, urological-gynaecological, abdominal and traumatological surgery.  Conducted comparisons with other haemodynamic monitoring devices to determine cardiac output showed a good correlation of the measured values between the methods.  The method of advanced haemodynamic monitoring by the LiDCOrapid was tested against validated methods in intensive care medicine as well as for intraoperative use (studies appendix 2). It showed a good correlation during different haemodynamic situations against established methods. But in contrast to the oesophageal Doppler there are no studies examining the influence of a LiDCOrapid driven algorithm on hospital length of stay.  The measurements of liver function should be performed by the established ICG-Clearance as well as by the newly developed LIMAX-Test. This technique is based on the kinetic analysis of an administered intravenous bolus of 13C-Methacetin (2ml/kg KG).  The chemical master reaction is the demethylation by the CYP1A2 in the liver. The formed 13CO2 will be eliminated by respiration and measured online by a non-dispersive infrared analyzer directly on the bedside and therefore offers a direct value of the metabolic capacity of the liver . Both methods are established methods of the Department of general, visceral and transplantation surgery of the Charité – Universitaetsmedizin Berlin, Campus Virchow Clinic for the pre- and postoperative evaluation of patients undergoing liver resection surgery and are correlated during the clinical course to the sonography of the liver vessels and MRI exams of the liver. |
| 10. Description of the intended procedures/ examination methods and the eventual change in usual medical practice which is anticipated (what is the ‘routine’ and what will be done differently from this in the trial?) | The perioperative care of all patients is conducted according to the published and certified (according to DIN EN ISO 9001) standard operating procedures of the Department of Anaesthesiology and Intensive Care Medicine.  Except the additional non-invasive measurement of the tissue oxygenation and of the transcranial Doppler there will be no study-related adaption of the clinical procedures or examinations. That includes preparation for anaesthesia, the induction and maintenance as well as the postoperative monitoring in the post-anaesthesia care as well as in the intensive care unit and the indicated invasive monitoring of the patients. The blood exams will be performed according to routine clinical procedures of the involved clinical departments. The blood values are analysed scientifically in collaboration with Dr. Weimann of the central institute of laboratory medicine and pathobiochemistry at the Campus Virchow Clinic but without that there are additional blood samples taken. Independently of the study protocol laboratory values are examined by the Department of general, visceral and transplantation surgery to determine the liver regeneration and the liver function. During the conductance of the study blood samples are taken only for scientific interests regarding the perioperative immunological and endothelial function. Discharge from the post-anaesthesia care or the intensive care unit will be indicated according to the discharge criteria by the Aldrete score (see appendix 9 and 10).  After admission in the Department of general, visceral and transplantation surgery clinical routine exams according to the clinical path for liver resections are performed. Patients are screened for the inclusion and exclusion criteria and after acceptance the questionnaire of quality of life (EQ-5D, see appendix 8, equally at the end of the study), the CAGE and AUDIT questionnaire for the determination of alcohol used disorders are completed with the patients. The patients are motivated to drink fluids up to two hours prior to induction of anaesthesia.  During the induction of anaesthesia only less than 200 ml of a balanced crystalloid infusion will be administered to assure the administration of the anaesthesia drugs via the intravenous line.  For the coverage of blood pressure drops during the induction of anaesthesia the blood pressure will be determined every minute up to the establishment of the oesophageal Doppler. The non-invasive measurement of the tissue oxygenation and the transcranial Doppler will be performed throughout surgery. In the case of a short-term hypotension the administration of Effortil 2 ml i.v. and in prolonged periods of hypotension the continuous administration of norepinephrine to maintain target blood pressure values are indicated.  After positioning of the patient in the theater and establishment of the Doppler measurements in the Doppler group the volume protocol (see appendix 3) based on the values of advanced haemodynamic monitoring will be started. In the first group monitoring is based on the values of the oesophageal Doppler and in the second group based on the values of the LiDCOrapid monitor. In both protocol groups an initial administration of 200ml gelatin solution is performed. I the patient presents with signs of volume reagibility indicated by an increase of stroke volume, volume administration is repeated as long as stroke volume does not further increase. Subsequently target arterial blood pressure is reached according to the preoperative blood pressure values of the patients (see below) and a cardiac index higher than 2.5 l/min is maintained up to the end of the resection. After finishing the surgical manipulation of the liver optimization of cardiac preload by volume administration based on stroke volume is re-indicated.  In the control group measurements of the advanced haemodynamic values is performed by the oesophageal Doppler and the LiDCOrapid blinded for the present physicians in charge.  In the control group the administration of volume is performed according to the standards of the Department of anaesthesiology and intensive care medicine. An assessment of the volume requirements under consideration of the measureable parameters like blood pressure, heart rate, urine output, peripheral perfusion and derived parameter of an invasive arterial line, if it was indicated due to relevant comorbidities. In the control group the anaesthetist can administer vasopressors or positive inotropes according to his clinical experiences. The goal of the volume and circulatory therapy should be directed that the target blood pressure according to the preoperative values adapted to a normfrequent rhythm (heart rate between 40 and 100 beats per minute) is reached.  The patient should receive an intraoperative volume therapy by balanced crystalloids (e.g. Jonosteril ®) and unbalanced colloids (e.g. Gelafundin ®).  The definition of the target blood pressure (mean arterial pressure) derived from the preoperative values:  - Normotensive patients: > 70 mmHg  - Hypertensive patients: > 80 mmHg  - Hypotensive patients: > 60 mmHg  The surgery will be performed accordingt to the general clinical guidelines of the Department of general, visceral and transplantation surgery.  Every patient will be observed in an intensive care unit up to the point fulfilling the discharge criteria. Immediately after surgery routine blood samples are taken. If the patient is awake, has good adverse-effects reflexes and no nausea or vomiting and there is no surgical contraindication present, he can start with postoperative feeding with water ad libitum and subsequently with yoghurt and after a two hour tolerance solid foods.  The volume and fluid therapy during the postoperative course of the patient is conducted according to the clinical indications and experiences of the treating physicians of the involved departments. |
| 11. Evaluation and consideration of the forseeable risks and disadvantages to the the study participants in contrast to the expected benefit for them and future patients.  Risk-benefit evaluation) | The risks of using the oesophageal Doppler probe are seen as minimal. According to Deltex Medical the probe has thus far been used approximately 800,000 times and there has only once been a suspicion of an oesophageal injury. Previous studies have no indication of Doppler-associated complications. By avoiding nasal insertion of the probe one avoids the risk of epistaxis and for the purposes of this study oral insertion of the probe will be used.  Against these minimal risks stand the findings of previous studies using the oesophageal Doppler which have demonstrated a reduced rate of complications as well as a reduced length of ICU and hospital stay.  For the use of the LiDCOrapid there is no additionally risk for the study patients as the monitor derives the signal of the invasive arterial pressure measurement and calculates by algorithms the puls-power of the heart beats from the arterial blood pressure curve and determines consecutively stroke volume. Given that every patient gets an arterial line placed due to the kind of operation there is no additionally risk.  The development and the evaluation in the clinical context of a haemodynamic algorithm for liver resections on the basis of minimal-invasive monitoring is in due proportion to the risks of the participating patients. |
| a. Expected therapeutic benefit for the study participants (individual benefit for a single patient) | It has been shown in other patient populations that patients with improved haemodynamics suffered a lower rate of complications and had an earlier stabilisation of their clinical condition, thereby allowing them earlier to be discharged home.  This proven effect in other populations is also the benefit expected to be seen in the protocol guided groups of this trial. |
| b. Predicted future medical uses for patients (**Group benefit)** | It has to be the aim of this study to examine the influence of advanced haemodynamic monitoring and the use of clinical therapy protocols based on clinical studies to lower the rate of complications.  Given that this kind of surgery is common (approximately 250 interventions per year in the Department of General, Visceral and Transplantation sSurgery, Charité – University Medicine Berlin, Campus Virchow Clinic) and it is associated with a high rate of postoperative complications, the benefit of new knowledge is of high value for future patients to perform this intervention with a lower rate of complications. |
| c. Risks and discomfort for the study participants (list each seperately) | The risks of the oesophageal Doppler are injury to the oesophagus and bleeding in the nasopharyngeal cavity. The danger of an oesophageal injury can be classified as small given that, according to Deltex Medical, there has only been one case of a possible oesophageal injury in over 800,000 uses of the probe.  The danger of bleeding in the nasopharyngeal cavity is higher with nasal insertion of the probe. However as the probe will be inserted orally in this study then this risk can also be said to be small. Furthermore none of the previous studies attributed any relevant morbidity to the oesophageal probe.  Similarly the level of discomfort caused to patients is likely to be small. A nasogastric tube is comparable to a Doppler probe in size and shape. Positioning of nasogastric tubes is even tolerated well by awake patients using local anaesthesia. However in this study the oesophageal probe will be placed following induction of anaesthesia and removed again before extubation. Therefore the level of discomfort can be classified as minimum.  For the patients of the LiDCO group there is no additionally risk because the patients get an arterial line placed due to the kind of surgery. The signal of the arterial line will only be additionally analysed to determine the haemodynamic parameter.  The intended blood withdrawals will be performed in the same manner as the blood withdrawals due to the clinical routine. Generally by taking the blood samples there is no interference with the physical well-being. In isolated cases harmless disturbances of the circulation can occur that can be avoided substantially by taking a short-term rest after the blood withdrawals. At the point of puncture haematoma can occur. On rare occasions  At the site of the puncture the patient can get an hematoma. In rare cases injury of the blood vessels (veins and arteries) and nerves as well as inflammation of the site of the puncture can occur. A transmission of infectious diseases is not possible due to the use of sterile disposable systems and cannulas are used to withdraw blood probes. Additionally the trained staff aims to use intravenous lines to withdraw the blood probes. The amount of blood withdrawn will be approximately 20-30 ml per day during the five postoperative days. |
| 12. Risk control measures | The dangers of placing the oesophageal probe comprise oesophageal injury and nasopharyngeal bleeding. For this reason pre-existing pathology or recent operations in the area of the oesophagus or nasopharyngeal cavity represent risk factors for these complications and should be regarded as contraindications to study participation.  The siting of the oesophageal probe takes place under the strict supervision of an anaesthetist and all patients will be monitored post-operatively until their discharge or for 30 days. |
| 13. Study termination criteria | Under the following conditions premature separating of a patient from the study occurs in accordance with the abort criteria:  The following events can be regarded as reasons for termination:  • personal wish of the patient  • every other situation, where in the opinion of the study physician, further participation in the trial would not be in the best interests of the patient  • unblinding in the case of a medical emergency  • significant protocol breaches  • Subsequent occurrence of an exclusion criterion  A premature cessation of the study and/or an abandonment of the entire study can occur in the following circumstances:  • Decision of the study leader where untenable risks exist according to risk-benefit analysis  • new (scientific) developments during the course of the study demonstrating a risk to participants by further continuation of the study (a positive risk-benefit ratio is no longer applicable) |
| 14. Number, age and sex of participants | There are no studies in the literature for the primary and secondary endpoint that show reliable data to enable a statistical calculation of the number of patients that would be needed.  Therefore this study is planed as a pilot study including 20 patients per group that means overall 60 patients are included. In this study no gender-based selection of the study patients will be performed. Man and woman aging more than 18 years are included in the study. |
| 15. Description of statistical methods and biometric basis for required number of cases with signature of statistician | For all endpoints of the study explorative data analyses will be performed and descriptively analysed. Because of the small sample sizes and possible outliers and/or skew distributions, the primary and secondary endpoints will be analysed using non-parametric statistical tests (qualitative data: Fisher’s exact test, quantitative data: Mann-Whitney-U test for independent groups or Wilcoxon-test for paired observations).  All test will be performed with an alpha error = 5% (two-sided). The calculated p values will be considered as explorative and do not allow confirmative generalization. Due to the same reason we do not perform alpha adjustment for multiple testing.  As we can not perform a calculation of the sample size the number of patients to be included in this study follows objective considerations and the clinical possibilities to perform the conductance of this pilot study. With the results of this study a consecutive prospective randomized study with calculation of the sample size shall be initiated.  The analysis of the study results will be performed according to the “intention-to-treat” as well as to the “per-protocol” principle.  The numerical calculations are performed with the statistical program SPSS, Version 17, Copyright© SPSS, Inc., Chicago, Illinois 60606, USA. |
| 16.  a. Statement of exclusion criteria, with explanation if necessary | **Inclusion criteria:**   - Written informed consent from patient - Patients undergoing elective liver resection in Charité - University Medicine Berlin, Campus Virchow-Clinic   **Exclusion criteria:**   - Aged less than 18 years - No written informed consent from patient - For female patients: pregnancy or lactation - Inability to communicate freely in the German language - Lack of willingness to safe and hand out pseudonymised data within the clinical study - Simultaneous participation of the patient in another study - Accommodation in an institution due to an official or judicial order - Members of staff of the Charité - Unclear history of alcohol used disorder - Advanced disease of the oesophagus of nasopharyngeal cavity - Operations in the area of the oesophagus or nasopharynx within the last two months - History of bleeding tendency e.g. Von Willebrands disease - Neurological or psychiatric disease - Chronic heart failure New York Heart Association (NYHA) class IV - American Society of Anaesthesiologists (ASA) classification greater than IV - Chronic renal failure with dependency of haemodialysis - Existence of a pulmonary oedema in the pre-operative chest x-ray - History of intracranial haemorrhage within one year - Allergy to gelatin |
| 16.  b. **Participant information** *(who gives this statement verbally and indicates how much time remains between declaration and consent, otherwise by reference to their contents in an appendix* | see appendix |
| c. **Declaration of consent** *(reference to their contents in an appendix is possible)* | see appendix |
| d. If necessary **obtain information and consent from legal representatives** *( also if required describe the procedure to obtain a judicial review)* | Not applicable |
| 17. Methods to recruit study participants (notices, newspaper advertisements, etc) | All patients undergoing abdominal surgery in the Charite Campus Virchow will be screened to see if they fulfil the inclusion criteria. |
| 18. If necessary: **Reason for inclusion and justification of therapeutic use in individuals who are under-age and/or are incapable of giving consent** | Not applicable. |
| 19. Relationship between the study participants and the research physician (is the treating physician also the research physician?) | The patients will form part of the daily treatment routine within the departments of General, Visceral and Transplantation Surgery and Anaesthesiology and Intensive Care in the Charite Campus Virchow clinic. The study physicians themselves will also be recruited from these same departments. Both departments have extensive experience with clinical studies.  Patients can decide freely whether or not to enter the study and their decision on whether or not to participate will not have an influence on their quality of care.  The study staffs are not the treating doctors. |
| 20. If applicable: Ifinclusion of dependant individuals is possible the research physician, or sponsor, must give an explanation | Not applicable. |
| 21. Outline measures which allow study participants to make a statement on whether they’re participating simultaneously in other studies or before the expiry of a period specified in earlier studies | At the time of the entry assessment the study participant will be asked if he/she is participating in a parallel study. If he/she is participating in another study, or has participated in another study within the past week before inclusion, then he/she cannot participate according to the exclusion criteria set for this study. |
| 22. If applicable: Remuneration and/or reimbursement of costs of study participants (amount, specify expenses paid) | Not applicable. |
| 23. If applicable: Plan for further care and medical support of the affected individuals after termination of the study | The study duration is limited from the beginning of surgery up to the first postoperative day. After the operation a monitoring period follows until hospital discharge or until day 8 postoperatively whichever is earlier. The postoperative care of the patient is ensured as the patient will only be discharged from the hospital after fulfilment of the discharge criteria. That offers post-trial coverage to aim for avoidance that the patient does suffer unrecognized complications.  The study will be unblended after the last visit of the last patient. |
| 24. If applicable: Insurance of the study participants (insurance confirmation and conditions of insurance, insurer, coverage, duration of insurance) | The ethical application, the participant information and the declaration of consent were sent by email to Ms. E. Eckert to receive the confirmation of the hospital insurance for this study. |
| 25. If applicable: Documentation procedure (reference to the CRF-**records** if possible) | The data acquisition will be accomplished by means of an paper-based case report form.  The data will be saved under a pseudonym. The study personal generates for every newly registered patient a pseudonym according to the below described guidelines. The categorical allocation of each patient is kept on a paper record which must be stored securely.  The pseudonym will be generated from the first two letters of the study name, the number of the center CVK=1, CCM=2, and a three digits incrementally increasing number.  As an example for the first study patient:  LE 1 001 |
| 26. If applicable: A description of the state of health of any healthy individuals concerned must be documented | Patient inclusion occurs by virtue of their having disease that indicates a liver resection. No healthy patients will be included in this study. |
| 27. If applicable: Methods to detect, document and communicate adverse events (when, with whom and how??) | The patients will be supervised intensively during the operation by the anaesthetist and can only leave the operation area when the discharge criteria are fulfilled in the recovery room.  Apart from the routine daily care delivered by the physicians in the departments of General, Visceral and Transplantation Surgery, there will also be a visit from the study physicians of the Anaesthesiology department, so that adverse events can be detected.  The occurrence of adverse events should be communicated to the study director within 24 hours.  A liver resection represents a surgical intervention with increased risk and deviation of parameter frequently occurs. Due to that fact a list of exceptions will be generated because the deviations of the values are related to the surgical intervention and are not indicative of an adverse event induced by the study protocol. For the values of perioperative blood samples a plausibility list will be generated which regulates the limits the pathological values from the reportable events.  Post-operative monitoring will continue until the day of hospital discharge or the 8th post-operative day, whichever is earlier. |
| 28. Procedures to ensure the secrecy of stored data, documents and, if applicable, samples. State how the encoding of patient data occurs *(please don’t use patient initials and birthdays as coding schemes!)* | A pseudonym is generated for every patient and the list of pseudonyms is kept locked away. Patient documentation is only done using the pseudonym.  The pseudonym will be formed from the first two letters of the study name followed by the number of the center CVK=1, CCM=2, followed by a three digits incrementally rising number.  As an example for the first patient of the study:  LE 1 001 |
| 29. Explanation of maintenance of data security | The study physician will use the personal data for the purpose of administration and execution of the study, as well as for the purpose of research and statistical analysis.  In the event of any transfer of data about study participants a code number must be used (the data is recorded as a pseudonym). Only the study physician and her co-workers can have access to the code key which allows a connection to be made between the patient and the study related data.  All records through which the patient can be identified must be treated with strict confidentiality. |
| 30. Names and addresses of the organisation conducting the study as the study center or the study laboratory, as well as the study director and study physicians | Prof. Dr. Claudia Spies  Dr. Aarne Feldheiser  Ansgar Jones  Dr. Nikola Magheli  André Haas  Dr. Vera von Dossow  Dr. Christian Pille  Universitätsklinik für Anästhesiologie und operative Intensivmedizin,  Charité – Universitätsmedizin Berlin,  Charité Campus Mitte  Charitéplatz 1, 10117 Berlin  Charité Campus Virchow Klinikum  Augustenburger Platz 1, 13353 Berlin  Tel.: +49 (0) 30 450 551 001 / 12  Fax: +49 (0) 30 450 551 900  E-Mail: [anaesthesie-virchow-klinikum@charite.de](mailto:anaesthesie-virchow-klinikum@charite.de)  Prof. Dr. Peter Neuhaus,  PD Dr. Ulf Neumann  Dr. M. Stockmann  Dr. M. Bahra  Klinik für Allgemein-, Visceral- und Transplantationschirurgie  Charité – Universitätsmedizin Berlin  Charité Campus Virchow Klinikum  Augustenburger Platz 1, 13353 Berlin  Tel.: +49 (0) 30 450 55 20 01  Fax: +49 (0) 30 450 55 29 00  E-Mail: ulf.neumann@charite.de  Dr. med. Andreas Weimann  Zentralinstitut für Laboratoriumsmedizin und Pathobiochemie  Campus Virchow-Klinikum  Augustenburger Platz 1, 13353 Berlin  Tel.: +49-30-450 569032  Fax: +49-30-450 569900  E-Mail: andreas.weimann@charite.de |
| 31. Statement regarding the organisation of the study center, particularly its suitability in terms of having the resources and personnel to execute a clinical study, as well as its experience in conducting similar studies previously | The Charité – Campus Virchow-Klinikum is a maximally equipped hospital with a 24 hour capability for emergency patient care. The departments of General, Visceral and Transplantation Surgery and Anaesthesiology both possess extensive experience in the execution of clinical studies. |
| 32. Agreement regarding access of the examiner/chief examiners/directors of the clinical examination to the data and the principles concerning this publication | The recording of patient data will occur in a strictly confidential manner and any transfer of patient data will be via the use of pseudonyms.  The research team has only access to the personal data insofar as it is necessary for them to conduct the study. The ERAS database is a web-based eCRF.  The publication is intended for a peer-reviewed journal independent of the final result. |
| 33. Statement regarding financing of the study (we refer to § 263 StGB)  a. Source of finance (name and position) | Charité – Universitätsmedizin Berlin  Funding by the internal university promotion. |
| b. Calculated costs per participant, and in total | The oesophageal Doppler probes will cost approximately 120,00 €, the card for the use of the LiDCOrapid will be provided by LiDCO Group Plc. The cerebral oxymeter will be provided by Somanetics Corporation and the InSpectra StO2-Sensoren will be provided by Hutchinson Technology.  Laboratory costs amount to approximately 60,00€ per participant |
| c. Amount of reimbursement per participant and in total | No reimbursement of participants |

Name and signature of the applicant:

I hereby confirm that the information contained in this application is correct. I am of the opinion that the above mentioned study can be performed in compliance with the protocol national legal regulations.

I confirm that I am acting in accordance with the §19 Berlin Data Protection Act (BlnDSG) regarding the automated processing of person specific and person traceable data and that I will make available to the Data Protection Commissioner of the Charite a file and process description in accordance with §19a.

I am aware that in accordance with §5 BlnDSG, if a situation arises where data subject to professional confidentiality must be processed (e.g. medical pledge of secrecy), then I must apply beforehand to the Data Protection Commissioner at the Charite for permission, and only on getting permission may I use the procedure

Name: Prof. Dr. med. Spies

Vorname: Claudia

Adress: Augustenburger Platz 1

Position: Director of the Department of Anaesthesiology und Intensive Care Medicine, Charité – Campus Mitte and Campus Virchow-Klinikum

Place, Date: Berlin, the

Signature:

**References:**

1. Bechstein WO, Neuhaus P (2000) [Bleeding problems in liver surgery and liver transplantation]. Chirurg 71: 363-368.

2. Chen H, Merchant NB, Didolkar MS (2000) Hepatic resection using intermittent vascular inflow occlusion and low central venous pressure anesthesia improves morbidity and mortality. J Gastrointest Surg 4: 162-167.

3. Johnson M, Mannar R, Wu AV (1998) Correlation between blood loss and inferior vena caval pressure during liver resection. Br J Surg 85: 188-190.

4. Jones RM, Moulton CE, Hardy KJ (1998) Central venous pressure and its effect on blood loss during liver resection. Br J Surg 85: 1058-1060.

5. Riecke B, Neuhaus P, Stockmann M (2005) Major influence of oxygen supply on 13CO2:12CO2 ratio measurement by nondispersive isotope-selective infrared spectroscopy. Helicobacter 10: 620-622.

6. Mythen MG, Webb AR (1995) Perioperative plasma volume expansion reduces the incidence of gut mucosal hypoperfusion during cardiac surgery. Arch Surg 130: 423-429.

7. Sinclair S, James S, Singer M (1997) Intraoperative intravascular volume optimisation and length of hospital stay after repair of proximal femoral fracture: randomised controlled trial. BMJ 315: 909-912.

8. Gan TJ, Soppitt A, Maroof M, el-Moalem H, Robertson KM, et al. (2002) Goal-directed intraoperative fluid administration reduces length of hospital stay after major surgery. Anesthesiology 97: 820-826.

9. Venn R, Steele A, Richardson P, Poloniecki J, Grounds M, et al. (2002) Randomized controlled trial to investigate influence of the fluid challenge on duration of hospital stay and perioperative morbidity in patients with hip fractures. Br J Anaesth 88: 65-71.

10. McKendry M, McGloin H, Saberi D, Caudwell L, Brady AR, et al. (2004) Randomised controlled trial assessing the impact of a nurse delivered, flow monitored protocol for optimisation of circulatory status after cardiac surgery. BMJ 329: 258.

11. Wakeling HG, McFall MR, Jenkins CS, Woods WG, Miles WF, et al. (2005) Intraoperative oesophageal Doppler guided fluid management shortens postoperative hospital stay after major bowel surgery. Br J Anaesth 95: 634-642.

12. Noblett SE, Snowden CP, Shenton BK, Horgan AF (2006) Randomized clinical trial assessing the effect of Doppler-optimized fluid management on outcome after elective colorectal resection. Br J Surg 93: 1069-1076.

13. de Wilde RB, Schreuder JJ, van den Berg PC, Jansen JR (2007) An evaluation of cardiac output by five arterial pulse contour techniques during cardiac surgery. Anaesthesia 62: 760-768.

14. Missant C, Rex S, Wouters PF (2008) Accuracy of cardiac output measurements with pulse contour analysis (PulseCO) and Doppler echocardiography during off-pump coronary artery bypass grafting. Eur J Anaesthesiol 25: 243-248 Epub 2007 Nov 2009.

15. Costa MG, Della Rocca G, Chiarandini P, Mattelig S, Pompei L, et al. (2008) Continuous and intermittent cardiac output measurement in hyperdynamic conditions: pulmonary artery catheter vs. lithium dilution technique. Intensive Care Med 34: 257-263 Epub 2007 Oct 2006.

16. Kim JJ, Dreyer WJ, Chang AC, Breinholt JP, 3rd, Grifka RG (2006) Arterial pulse wave analysis: An accurate means of determining cardiac output in children. Pediatr Crit Care Med 7: 532-535.

17. Pittman J, Bar-Yosef S, SumPing J, Sherwood M, Mark J (2005) Continuous cardiac output monitoring with pulse contour analysis: a comparison with lithium indicator dilution cardiac output measurement. Crit Care Med 33: 2015-2021.

18. Hamilton TT, Huber LM, Jessen ME (2002) PulseCO: a less-invasive method to monitor cardiac output from arterial pressure after cardiac surgery. Ann Thorac Surg 74: S1408-1412.

19. Levy MM, Fink MP, Marshall JC, Abraham E, Angus D, et al. (2003) 2001 SCCM/ESICM/ACCP/ATS/SIS International Sepsis Definitions Conference. Crit Care Med 31: 1250-1256.

20. Bernard GR, Artigas A, Brigham KL, Carlet J, Falke K, et al. (1994) Report of the American-European Consensus conference on acute respiratory distress syndrome: definitions, mechanisms, relevant outcomes, and clinical trial coordination. Consensus Committee. J Crit Care 9: 72-81.

21. Bellomo R, Ronco C, Kellum JA, Mehta RL, Palevsky P (2004) Acute renal failure - definition, outcome measures, animal models, fluid therapy and information technology needs: the Second International Consensus Conference of the Acute Dialysis Quality Initiative (ADQI) Group. Crit Care 8: R204-212.

**Appendix 1:**

**Selection of relevant outcome studies using the Oesophageal doppler:**

| **Study** | **n-number**  **ASA-Status** | **Surgical specialty** | **Patient outcomes** |
| --- | --- | --- | --- |
| Mythen 1995 | 60  ASA III | Cardiac surgery | No increase in morbidity  41% reduction in ICU care  37% reduction in hospital care |
| Sinclair 1997 | 40  ASA II/III | Hip fractures  Traumatology | 39% reduction in hospital care |
| Gan 1999 | 44 | Urology/  Gynaecology | 33% reduction in hospital care |
| Venn 2002 | 90  ASA III/IV | Hip fractures  Traumatology | 44% reduction in time till medically fit  23% reduction in hospital care |
| McKendry 2000 | 39 | Cardiac surgery | 19% reduction in hospital care |
| Gan 2002 | 100  ASA I/II/III | Urology/  Gynaecology | 29% reduction in hospital care |
| Wakeling 2005 | 134  ASA I-III | Abdominal  surgery | 37 % reduction in patient complications  13 % reduction in hospital care |
| Noblett 2006 | 108 | Abdominal-surgery | 22 % reduction in hospital care  Reduction in life-threatening complications |

**Abbreviations:**

ASA American Society of Anesthesiologists

ICU Intensive care unit

**Appendix 2:**

|  | | | **Differences to control method** | | | **Limits of Agreement** | |
| --- | --- | --- | --- | --- | --- | --- | --- |
| **Studies and control method** | **No. Pat.** | **No.**  **Obs.** | **Bias**  **L/min** | **Precision**  **L/min** | **95% Co.-In.**  **L/min & %** | **Upper**  **L/min** | **Lower**  **L/min** |
| de Wilde et al.  Cardiac surgery, intra-op.  CO-thermodilution | 27  88 | 199  301 | -0.17  -0.02 | 0.69  0.65 | 1.38 (28.6%)  1.3 (25.7%) | -1.55  -1.28 | 1.20  1.32 |
| Missant et al.  Cardiac surgery, intra-op.  CO-thermodilution | 20 | 149 | -0.03 | 0.65 | 1.3  (29%) | -1.33 | 1.26 |
| Costa et al.  Liver tranpslantation, post-op.  CO-thermodilution | 23 | 151 | 0.29 | 1.09 | 2.17  (16.8%) | -1.87 | 2.46 |
| Kim et al.  Pediatric intensive care unit (*: cardiac index)  CO-thermodilution | 20 | 73 | 0.19* | 0.14* | 0.28*  (8.5%) | -0.09* | 0.47* |
| Pittman et al.  Intensive care unit (24 hrs.)  Lithium-thermodilution | 21 | 83 | -0.01 | 0.82 | 1.64  (27%) | -1.65 | 1.63 |
| Hamilton et al.  Cardiac surgery, post-op.  Lithium-thermodilution | 20 | 80 | 0.1 | 0.6 | 1.2  (21.8%) | -1.1 | 1.3 |

**Abbreviations:**

No. Number

Pat. Patients

Obs. Exams

Ko.-In. Confidence-Intervall

**Appendix 3:**

**Algorithm for the goal-directed volume and catecholamine therapy of the intervention groups**

*: The stroke volume after the initial preload-optimization is the Starting-SVI.

^#^: The drop of the stroke volume index is related to the Starting-SVI.

^x^: Are there clinical (a opinion of the surgeon in combination with the estimation of the anaesthesists) or haemodynamically (in the form of an elevated central-venous pressure in combination with the clinical estimation) sign for a backward failure of the right ventricle to the liver, the administration of enoximone (initial dosis: 2,5µg/kg/min, can be increased up to 10µg/kg/min or a bolus administration of 1µg/kg) or nitroglycerine should be indicated according to the clinical estimation of the anaesthesists in charge. The simultaneous administration of both drugs must not be indicated in the same patient due to the risk of hypotension.

**Abbreviations of the diagram:**

SVI Stroke volume index

SBP Systolic arterial blood pressure

MAP Mean arterial blood pressure

**Appendix 4**

**Definition of infectious complications:**

For the diagnosis of an infection the following SOP from our hospital will be applied in accordance with the CDC criteria and ATS guidelines [15]:

**Pneumonia:**

In this case one of the following criteria must be fulfilled:

**Crackles on auscultation or dullness to percussion**

and one of the following signs:

- Newly developed purulent sputum or a change in its characteristics,
- Microbes isolated from blood cultures.
- Pathogen isolated from bronchoalveolärer lavage, bronchial swab, transtracheal aspirate or biopsy sample

**Chest x-ray demonstrating new or progressive infiltrates, opacification, cavitation or a pleural effusion.**

and one of the following signs:

- Newly developed purulent sputum or a change in its characteristics,
- Microbes isolated from blood cultures.
- Pathogen isolated from bronchoalveolärer lavage, bronchial swab, transtracheal aspirate or biopsy sample

**Tracheobronchitis:**

2 of the following criteria must be met without signs of pneumonia:

- Fever (38°C)
- Cough
- New or increased sputum production
- Dry crackles

and **one** of the following criteria must be met:

- Microrganisms isolated through culturing tracheal secretions or material obtained via bronchoalveolar lavage.
- Positve antigen-test from the airway

**Wound infection:**

**Superficial post-operative wound infection:**

Infection at the incision site within 30 days of operation which only involves the skin or subcutaneous tissues,

and one of the following criteria are met:

- Purulent discharge from the superficial incision
- Microbe isolated from culture of an aseptically obtained wound secretion or a tissue culture of the superficial incision

and one of the following signs:

- Pain
- Tenderness
- localised swelling
- Erythema or warmth
- The surgeon would open the superficial incision except that a **negative culture exists before the diagnostic decision**.

**Deep post-operative wound infection:**

Infection within 30 days of operation. The infection appears to be associated with the operation and involves muscle tissue and fascial layers.

In addition to this one of the following criteria must be met:

- Purulent secretions from deep in the incision but not from an organ and/or body cavity
- Spontaneous drainage or deliberate surgical incision when the patient has at least one of the following symptoms:
- Fever (>38°C)
  - localised pain or tenderness
  - culture is confirmed negative
- An abscess or other sign of infection is evident from clinical, radiological or histopathological examinations or during re-operation
- diagnosis made by the surgeon

**Urinary tract infection:**

The patient has at least **one** of the following signs or symptoms with no other recognized cause:

- Fever (>38°C)
- Urgency
- Frequency
- Dysuria or suprapubic tenderness

and the patient has a positive urine culture, that is, ≥ 10^5^ microorganisms per cm^3^ of urine with no more than two species of microorganism.

The patient has at least **two** of the following signs or symptoms with no other recognized cause:

- Fever (>38°C)
- Urgency
- Frequency
- Dysuria or suprapubic tenderness

And at least **one** of the following:

- Positive dipstick for leukocyte esterase and/or nitrate
- Pyuria (urine specimen with ≥ 10 WBC/mm^3^ or ≥ 3 WBC/high power field of unspun urine)
- Organisms seen on Gram stain of unspun urine
- At least ***two*** urine cultures with repeated isolation of the same uropathogen (gram-negative bacteria or *S. saprophyticus*) with ≥ 10^2^ colonies/mL in nonvoided specimens
- ≤ 10^5^ colonies/mL of a single uropathogen (gram-negative bacteria or *S. saprophyticus*) in a patient being treated with an effective antimicrobial agent for a urinary tract infection
- Physician diagnosis of a urinary tract infection
- Physician institutes appropriate therapy for a urinary tract infection

**Sinusitis:**

Sinusitis must meet at least one of the following criteria:

- Patient has organisms cultured from purulent material obtained from sinus cavity
- Patient has at least ***one*** of the following signs or symptoms with no other recognized cause:
  - Fever (≥38°C)
  - Pain or tenderness over the involved sinus
  - headache, purulent exudate, or nasal obstruction
- *And* at least ***one*** of the following**:**
  - Positive transillumination
  - Positive radiographic examination

**SIRS:**

Definition of sepsis according to the criteria of the ”Society of Critical Care Medicine Consensus Conference“ :

At least ***two*** of the following criteria must be fulfilled for the diagnosis of SIRS:

- Body core temperature > 38,0 °C or < 36,0 °C
- Heart rate: >90/min without ß-antagonism
- Or ***two*** of the following signs of respiratory failure:
  - Respiratory rate: < 20/min
  - Hyperventilation P_a_CO_2_ < 32mmHg in spontaneous breathing patients
  - p_a_O_2_ < 70 mmHg (spontaneous breathing patients)
  - p_a_O_2_/F_i_O_2_ < 175 (in mechanically ventilated patients and without medical history)
- WBC count: >12000/mm^3^ or <4000/mm^3^ or >10% immature forms

**Sepsis:**

- SIRS-Criteria (see above) are fulfilled
- Infection, documented or suspected

As the case may be

- An excessive activation of primarily protective defensive systems of the septic patient in terms of a host defense failure disease

**Severe Sepsis:**

Sepsis-Criteria and systemic toxicity or decreased organ perfusion **with two or more** characteristic signs:

- - Acute renal failure (Creatinine increase > 0.5 mg/dl or Oliguria < 0.5 ml*kg^-1^*h^-1^)
  - Increased plasma lactate (>1.8 mmol/l)
  - CI >4.0 l/min with SVR <800 dyn*s*cm^-5^
  - Metabolic acidosis (pH <7.3 or base deficite >5)
  - Arterial hypoxemia: P_a_O_2_ <75 mmHg (<10kPa)
  - Decrease of platelets within the last 24h with no other recognized cause (<100000/ml or decrease of more than 50% of the initial value)
  - Coagulation abnormalites within the last 24h with no other recognized cause (INR >1.5 or aPTT >1.2 * initial value)
  - Altered mental status with a sudden decrease of the Glascow-Coma-Scale

**Septic Shock:**

Signs of sepsis and refractory arterial hypotension despite adequate fluid resuscitation with decreased tissue perfusion or organ dysfunction

Hypotension is defined by at least one of the following characteristics:

- - Systolic blood pressure < 90 mmHg
  - Lasting decrease of blood pressure < 40 mmHg despite adequate fluid resuscitation without antihypertensive drugs
  - Therapy with vasopressors to maintain blood pressure < 90 mmHg

**Definition of ARDS according to the american-european consensus-conference (AECC) :**

- acute onset of the disease
- paO_2_/FiO_2_ (Horovitz)-Index < 200 mmHg independent of the applied PEEP
- bilaterale infiltrates in a.p.-x-ray of the thorax
- PCWP < 18 mmHg 🡺 exclusion of a cardial edema, where required using an esophageal echocardiography

**Lung Injury Score (LIS) by Murray (1988):**

- Goal of the score is the rating of the pulmonary function, i.e. in ARDS
- 4 criteria:
  - X-ray of the lungs
  - Number of quadrants showing alveolar consolidation on chest X-ray
    - 0 – 4 quadrants
  - Hypoxemia according to the Horovitz-Index:
    - > 300 mmHg
    - 225 – 299 mmHg
    - 175 – 224 mmHg
    - 100 – 174 mmHg
    - < 100 mmHg
  - PEEP:
    - < 5 cm H_2_O
    - 6 – 8 H_2_O
    - 9 – 11 H_2_O
    - 12 – 14 H_2_O
    - > 15 H_2_O
  - Compliance:
    - > 80 ml/cm H_2_O
    - 60 – 79 ml/cm H_2_O
    - 40 – 59 ml/cm H_2_O
    - 20 – 39 ml/cm H_2_O
    - < 19 ml/cm H_2_O

**Points:**

0 – 4

0

1

2

3

4

0

1

2

3

4

0

1

2

3

4

**Assessment:**

The sum oft he values divided by the number of groups evaluated:

Points:

0 No lung injury

0,1 – 2,5 slight or moderate lung injury

>2,5 severe lung injury

**Appendix 5**

**Definition of the RIFLE-Criteria of acute renal failure :**

According to: Bellomo R. et al., Critical Care 2004, 8:R204-R212

**Appendix 6**

**Definition of the Delirium Detection Score, the Nursing Delirium Rating Scale and the CAM-ICU for the detection of delirant states:**

**Delirium Detection Score = DDS**

| **Orientation** | # 0: Orientated to time, place and personal  identity, able to concentrate  # 1: Not sure about time and/or place, not able to  concentrate  # 4: Not orientated to time and/or place  # 7: Not orientated to time, place, and personal  identity |
| --- | --- |
| **Hallucinations** | # 0: Normal activity  # 1: Mild hallucinations at times  # 4: Permanent mild to moderate hallucinations  # 7: Permanent severe hallucinations |
| **Agitation** | # 0: Normal activity  # 1: Slightly higher activity  # 4: Moderate restlessness  # 7: Severe restlessness |
| **Anxiety** | # 0: No anxiety when resting  # 1: Slight anxiety  # 4: Moderate anxiety at times  # 7: Acute panic attacks |
| **Paroxysmal sweating** | # 0: No sweating  # 1: Almost not detectable, only palms  # 4: Beads of perspiration on the forehead  # 7: Heavy sweating |

**Nursing Delirium Rating Scale = Nu-DESC**

**CAM-ICU**

**
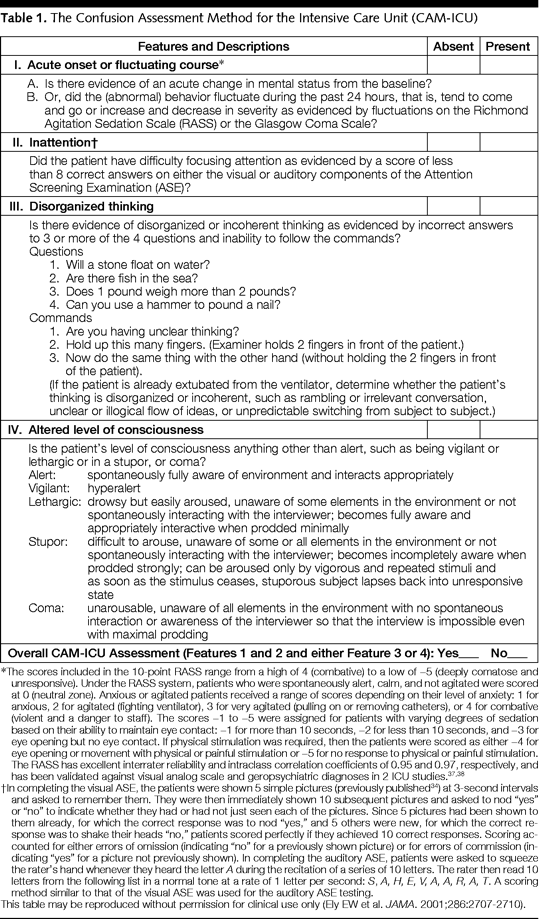
**

**Appendix 7:**

**Visual Analog Scala (VAS) / Numerical Rating Scale (NRS)**

- Necessary in all patients who can respond tot he question regarding pain
- Target pain level ≤ 3

| **VAS/NRS** | **Assessment** | **Points** |
| --- | --- | --- |
| Question about pain | No pain up to  maximal imaginable pain | 0 to  10 |
